# Implementation of the EU’s Health Technology Assessment regulation: where does existing methods guidance require concretization and what are the relevant methodological options?

**DOI:** 10.1017/S0266462324004793

**Published:** 2025-02-06

**Authors:** Gregor Goetz, Stefan Schandelmaier, Reinhard Busse, Claudia Wild, Dimitra Panteli

**Affiliations:** 1 Austrian Institute for Health Technology Assessment (AIHTA), Vienna, Austria; 2 Department of Health Care Management, Technische Universität Berlin, Berlin, Germany; 3 CLEAR Methods Center, Division of Clinical Epidemiology, Department of Clinical Research, University Hospital Basel and University of Basel, Basel, Switzerland; 4 School of Public Health, University College Cork, Cork, Ireland; 5 MTA–PTE Lendület “Momentum” Evidence in Medicine Research Group, Medical School, University of Pécs, Pécs, Hungary; 6 European Observatory on Health Systems and Policies, Brussels, Belgium

**Keywords:** health technology assessment, methodology, international collaboration, evidence standards, HTA regulation

## Abstract

**Objectives:**

The EUnetHTA Core Model^®^ is well-established in the HTA community. Some recommendations of corresponding guidance documents leave room for alternative methodological choices. Considering the new HTA regulation (HTAR), we aimed to identify needs for concretization (NCs) in EUnetHTA guidance and provide indicative methodological options.

**Methods:**

We carried out a qualitative document analysis and structured group discussion. Twenty-two EUnetHTA documents were screened using transparent criteria. Identified NCs were classified into topics according to the PRISMA statement and presented to Austrian HTA practitioners (*n* = 11) during a structured group discussion. Participants rated NC’s importance. To identify potential solutions, selected key handbooks for generic (Cochrane) and HTA-specific (IQWIG/NICE) evidence synthesis were systematically reviewed and matching content was charted against the NCs.

**Results:**

Thirty-two topics with varying numbers of NCs were identified, twenty-six during the screening process, and six from the group discussion. Most of the topics related to evidence synthesis methods (nine topics), evidence eligibility criteria (nine topics), risk of bias (three topics), and certainty assessment (three topics). Other topics related to information sources, search strategy, data collection process, data items, effect measures, and reporting bias. One or more methodological approaches and recommendations could be identified for each identified topic from the included methodological handbooks.

**Conclusions:**

Our analysis identified a need for concretization in some EUnetHTA guidelines. The structured overview of methodological options may support HTA doers in adapting and applying the guidelines to the national and local practical context.

## Introduction

The European Union adopted the Health Technology Assessment Regulation (HTAR) in January 2022 (1). The HTAR becomes legally binding in January 2025 and harmonizes collaboration and cooperation in evaluating medicinal products and medical devices; it is expected to provide a structure for conducting joint HTAs at the European level using state-of-the-art methods. The aim is to produce methodologically rigorous and timely scientific assessments supporting evidence-based decisions on the national level (2).

The European Network for Health Technology Assessment (EUnetHTA) has been supporting the scientific and technical cooperation and collaboration between HTA bodies (3): through developing methodological guidance and carrying out joint assessments for a range of technologies, EUnetHTA showcased that collaboration was feasible and could reduce redundancies (3).

The EUnetHTA Core Model^®^ – a methodological framework for both the production and sharing of HTA information – has become a cornerstone for international good practice. Since the creation of EUnetHTA in 2006, methodology guidelines on numerous topics have been continuously produced to guide HTA authors, especially in the context of methodological challenges (4). Following the adoption of the HTAR, to facilitate the implementation of the regulation, EUnetHTA’s latest project (EUnetHTA21) issued extensive further guideline documents, especially for joint clinical assessments (JCA) (5).

HTAR established the Member State Coordination Group on HTA (HTACG), with representatives from HTA bodies meeting regularly to coordinate and adopt joint HTA work. One of the HTACG’s tasks is to adopt methodological (and procedural) guidance by building on existing guidance documents, mainly from EUnetHTA21 (6).

Although the main methodological guidance relating to the production of systematic reviews (SR) is generally well established in EUnetHTA guidelines (3;5), some recommendations leave room to decide between alternative methodological options. This is not surprising, given the need to remain adaptive to the particularities of different technologies and to account for variability in the methodological approaches available and adopted by different HTA actors. For the HTAR to be implemented successfully, HTA bodies in different EU Member States need to contextualize and concretize specific methodological aspects for their national context. In this context, it is essential for national HTA agencies to both identify topics for which there exists a need for concretization and explore methodological options for these topics.

This article aims to describe identified methodological needs for concretization (NC) from the perspective of review authors working at the AIHTA and to summarize potential methodological solutions to identified needs.

## Methods

This qualitative study entailed a document analysis and a group discussion with SR authors from one national HTA institute. A study protocol is available online (Supplementary Appendix H).

### Step 1: Identification and screening of EUnetHTA guidance documents for needs for concretization

#### Eligible documents

We screened the EUnetHTA website for methodology guidance documents using the following eligibility criteria: Any document that (a) implicitly or explicitly entails methodological guidance to HTA authors and (b) can be applied to the assessment of effectiveness and safety. Screening was conducted from the perspective of HTA for non-drug interventions in hospitals. We focused on documents from the EUnetHTA methodology website (https://www.eunethta.eu/methodology-guidelines/) and EUnetHTA21 deliverables, including those related to the partial use of GRADE (see Supplementary Appendix D).

#### Screening

Twenty-two EUnetHTA documents were systematically screened for recommendations or by sections and sub-sections (if recommendations were not listed separately in the document). Methodological recommendations or other text passages describing methods were evaluated regarding clarity and specificity. We defined an NC if a guidance statement fulfilled one or more of the following criteria:

1. Guidance stating different alternative options instead of giving clear recommendation(s).
2. General lack of clarity concerning what is proposed within an EUnetHTA guidance.
3. The divergence between EUnetHTA guidelines and AIHTA methods (7) or standard practice at AIHTA.

#### Coding

One reviewer (GG) screened guidance recommendations. Text passages were coded as “clear guidance” or in “need for concretization” (binary coding) and further classified according to the three criteria highlighted above. We used atlas.ti (version 23.3.4) for qualitative data analysis.

Identified passages in NCs were clustered thematically using the labels and structure of the methods section of the Preferred Reporting Items for SRs and Meta-Analyses (PRISMA) statement (8).

### Step 2: Group discussion to refine needs for concretization and rate importance to HTA practitioners

A structured group discussion using the nominal group technique (9) with eleven researchers from the AIHTA was conducted to prioritize NCs among identified topics and determine additional ones, if relevant. All participants rated the importance on a scale from 1 to 6, with higher scores indicating higher relevance to the participants’ work practice.

### Step 3: Search for methodological solutions based on key methodological handbooks

The Cochrane Handbook (10) and methodological guides from the Institute for Quality and Efficiency in Health Care (IQWIG) (11) and the National Institute for Health and Care Excellence (NICE) (12) were screened for methodological options using targeted broad and specific keyword searches. Supplementary Appendix F lists all documents analyzed. Identified options were then charted against the NCs/topics identified from previous steps.

Text passages matching the scope of the identified NCs were extracted by one researcher (GG) and verified by another (DP or StS). Two researchers (GG, StS) independently rated whether the identified methodological options in the handbooks presented sufficiently clear methodological solutions to the respective NC (yes, somewhat, no). Discrepancies were resolved by consensus or by involving a third researcher (DP).

## Results

**Figure 1.**
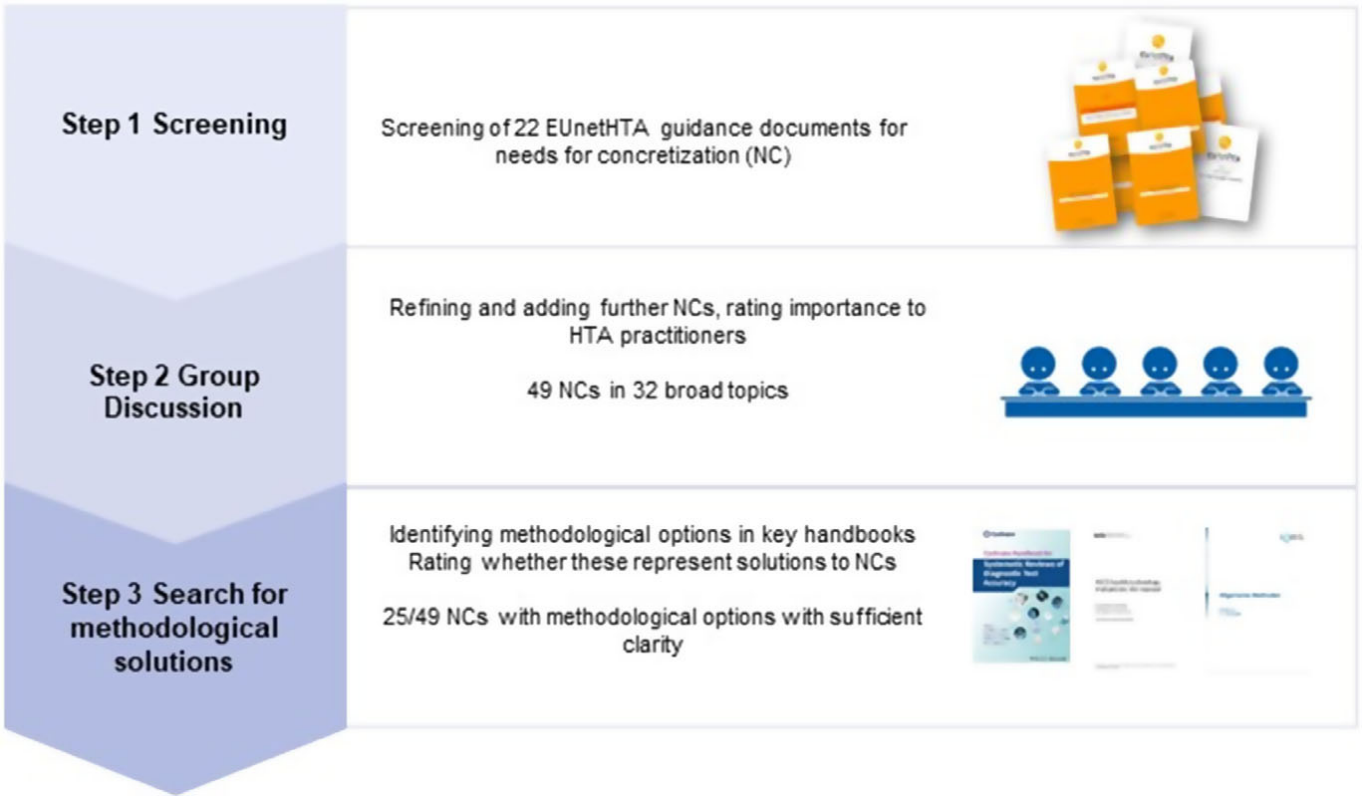
Overview of results of qualitative analysis of the need for concretization and identified potential solutions. NC, need for concretization.

The sections below summarize the results from our three-step approach: document screening, group discussion, and solution identification (Figure 1).

### Topics with identified needs for concretization and importance rating

Based on the screening of twenty-two eligible documents, thirty-six (out of 136) identified recommendations and forty-one further text passages related to NCs; the code tree of the qualitative content analysis is in Supplementary Appendix A. After grouping these text passages into NCs, broader methodological topics, and the group discussion with HTA practitioners, we defined forty-nine NCs in thirty-two methodological topics. These mainly covered evidence synthesis methods (nine topics), eligibility criteria (nine topics), risk of bias (RoB) assessment (three topics), and certainty assessment (three topics). Other topics pertained to information sources, search strategy, data collection process, data items, effect measures, and reporting bias (Table 1). Detailed information on identified NCs can be found in Supplementary Appendix C.

**Table 1.** Number of identified topics and needs for concretization

PRISMA-M domains	Topics	NCs
Eligibility	9	12
Information sources	2	2
Search	1	1
Data collection	0	0
Data items	2	2
RoB assessment	3	7
Effect measures	1	1
Synthesis methods	9	15
Reporting bias assessment	1	1
Certainty assessment	3	6
Other	1	2
Total	32	49

*Note:* PRISMA-M refers to the methods section of the PRISMA statement.

Abbreviations: NC, need for concretization; RoB, risk of bias.

Twenty-one topics included NCs important to AIHTA’s work, with a mean rating of 4+ (out of 6) or raised within the group input as important. Based on mean importance ratings, the most important NCs were found within the topics of eligibility of non-randomized studies of interventions (NRSI) and relating to RoB tools. Topics with NCs brought up by the group related to large number of RCTs, databases for search, additional information sources, reporting bias, formulating a recommendation, and patient/stakeholder involvement. An overview of NCs and team priority ratings can be found in Supplementary Appendix B.

### Overview of topics and methodological solutions to identified needs for concretization

In nineteen out of thirty-two topics, at least one methodological option of sufficient clarity (methodological solution) was identified for at least one need for concretization (NC) within the respective topic. For thirteen out of twenty-one important topics, the three handbooks provided methodological solutions to at least one NC within the following topics: Defining comparators, handling a large number of RCTs, databases, additional sources for searching, RoB Tools, handling studies with high RoB, handling subgroup analysis, indirect treatment comparison, missing data, reporting bias (incl. Sponsorship bias), value judgments, formulating a recommendation and patient and stakeholder involvement. Table 2 shows topics, NC counts, and availability of solutions from methods handbooks.

**Table 2.**
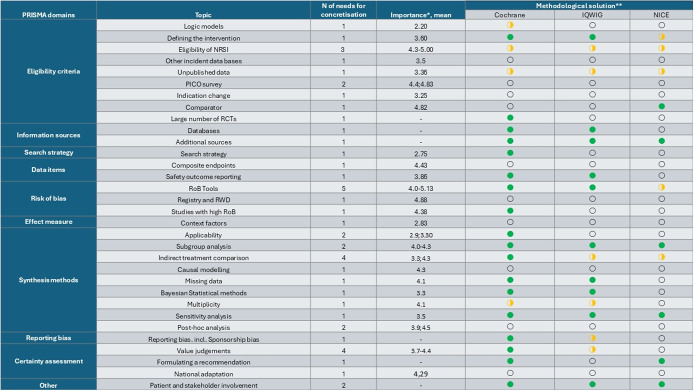
Overview of identified topics clustered according to PRISMA domains, number of needs for concretization, and methodological solutions

*Note:* Those NCs without a group rating were identified within the course of the group discussion and deemed as important to the AIHTA team.

Abbreviations: IQWIG, Institut für Qualität und Wirtschaftlichkeit im Gesundheitswesen; N, number; NICE, National Institute for Health and Care Excellence; NRSI, non-randomized studies of interventions; PRISMA, Preferred Reporting Items for Systematic Reviews and Meta-Analyses; RCT, randomized controlled trial; RoB, risk of bias.

* Importance rated between 1 and 6, higher values indicate higher relevance for HTA work practice.

** The topic-level rating was determined based on the presence of methodological solutions for at least one NC within the topic in question.

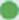
 Yes: Comprehensive guidance on at least one NC within a topic is provided that was judged to be a methodological solution to the respective NC.

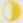
 Somewhat: The identified methodological options somewhat present a solution to the respective NC (e.g., mentioned, without providing the specific methodological solution to the identified NC).

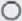
 No: Unknown, as methodological options were either not mentioned in the available guidance documents or too vaguely formulated to be regarded as a methodological solution.

### Methodological options based on key handbooks

The following sections present results based on the thirty-two topics, structured following the PRISMA methods section (8). The description of NCs and methodological options captures only topics for which there were relevant text passages for at least one NC in at least one handbook (see Supplementary Appendix E for complete list).

## Eligibility criteria

### Logic models

One NC related to whether logic models (e.g., analytical frameworks) should be routinely used within the scoping phase. Cochrane notes their benefit in documenting how interventions are intended to work and refining PICO questions, especially for complex interventions, aiding research question clarification and eligibility criteria definition.

### Defining the intervention

The decision on whether medical procedures or specific products are assessed represents another NC. Cochrane and IQWIG consider their reviews to be fundamentally based on healthcare interventions, not individual products. NICE states that their reviews are based on technologies.

### Eligibility of non-randomized studies of interventions (NRSI)

Three NCs addressed NRSI and minimum design features. Cochrane mentions two main justifications for NRSI inclusion: RCTs only indirectly/incompletely address questions or are unfeasible (three further reasons are also noted). IQWIG prioritizes RCTs, requiring justification for including lower-level evidence (e.g., a dramatic effect). NICE states a strong preference for RCTs but recognizes NRSI can supplement or substitute RCTs in specific situations (e.g., absence of RCTs or to contextualize RCT results).

### Search for and eligibility of unpublished data

One NC concerned how unpublished data, such as clinical study reports (CSR), should be incorporated. Cochrane recommends searching for unpublished data but notes the need for guidance on using regulatory sources. Both IQWIG and NICE consider unpublished data, including manufacturer CSRs.

### Specifying the comparator

One NC addressed whether certain aspects (e.g., economic considerations) can define and limit comparators. NICE committees select comparators using diverse criteria (e.g., NHS practice, cost-effectiveness) and may exclude cost-ineffective treatments after economic analysis.

### Reducing large numbers of RCTs

One NC addressed strategies to limit the number of studies to a feasible maximum. Cochrane recommends including all relevant RCTs but allows methodological or quality-based exclusion criteria (e.g., cluster/crossover RCTs, no placebo, unblinded outcome evaluation, short follow-up).

## Information sources

### Databases for search

One NC concerned minimum database requirements for journal publications (e.g., Medline) and data on clinical trials (e.g., clinicaltrials.gov) for routine searches. Cochrane mandates searching in general bibliographic databases (Medline and Embase) and CENTRAL. IQWIG recommends multiple databases, referring to the Cochrane Handbook. For focused searches in rapid reviews, at least two databases should be used.

### Additional information sources

One NC related to which further information sources or databases should be used depending on the topic and or research question. Cochrane provides several additional sources regarded as highly desirable to include in the search (e.g., reports, dissertations, conference abstracts) while checking reference lists of other reviews is considered mandatory. Other sources, such as CSR, are also mentioned as increasingly essential. IQWIG and NICE highlight the use of additional sources like those mentioned by Cochrane to identify additional relevant studies or documents depending on the research question.

## Search strategy

One NC related to whether a search strategy should be peer-reviewed. Cochrane strongly recommends peer review.

## Data items

### Disaggregation of composite endpoints

One NC related to the lack of clarity around how composite endpoints may be disaggregated, especially if reporting is poor. Cochrane states that composite safety measures may be disaggregated to describe distinct adverse events, without specifying methods when reporting is sparse. IQWIG suggests sensitivity analyses can exclude/include individual components from composite endpoints if sufficient data is available. Under appropriate conditions, individual endpoints can be determined and individually or newly re-calculated.

### Safety outcome reporting

One NC arose because EUnetHTA recommends using MedDRA terminology for safety reporting, which is not AIHTA’s current standard practice. Cochrane recognizes MedDRA as useful for collecting safety information and IQWIG acknowledges it.

## Risk of bias

### RoB assessment tools

Four NCs addressed which RoB tools to apply and how. An additional NC concerned assessing RoB for safety data. Cochrane recommends endpoint-level RoB assessment, specifying key tools for RCTs and observational studies with a control group, as well as for assessing the quality of safety assessment and reporting within included studies (see Table E6 in Supplementary Appendix E). There is no recommended approach for lower-level studies like single-arm trials. IQWIG uses its own RoB tool for RCTs, also citing relevant Cochrane tools. NICE mentions using validated tools without specifying which for different designs.

### Addressing high RoB

One NC referred to dealing with high RoB in identified trials. Cochrane considers it mandatory to include RCTs in the review if they meet pre-defined inclusion criteria, which can encompass quality considerations like blinding to allocation sequence. Cochrane’s latest RoB tool does not consider a study’s overall RoB but domain-specific and overall RoB per endpoint. In the description of the tool, Cochrane suggests subgroup analyses to assess whether effect sizes are possibly associated with RoB.

## Effect measures

### Context factors

One NC focused on how to incorporate context, like learning curves for therapeutic medical devices, in evidence synthesis. Cochrane suggests considering the wider context even for simple interventions, providing detailed guidance on intervention complexity (e.g., using logic models and subgroup analysis to account for context factors).

## Synthesis methods

### Applicability

Two NCs relate to addressing the applicability of identified evidence and whether and how data may be re-analyzed. Cochrane suggests that applicability should be addressed in the discussion and conclusion of a review using the GRADE approach (“indirectness”) (13). IQWIG highlights the importance of considering the applicability of findings, especially when considering the use of other SRs for HTAs.

### Subgroup analysis

Two NCs address potential criteria for considering, assessing, and reporting potential effect modification. All three methodological handbooks provide guidance on subgroup analysis, describing how these analyses can be done and highlighting challenges and pitfalls. All handbooks refer to a formal tool for assessing subgroup credibility (see Table E8 in Supplementary Appendix E).

### Indirect treatment comparison

Four NCs were identified around indirect treatment comparisons, with two addressing when to perform network meta-analyzes (NMA) and how to synthesize evidence from NMA. Two NCs focused on when to include or undertake naïve indirect comparisons and how evidence of such analyses should be assessed. Cochrane does not provide a clear decision rule on when to undertake NMA or other forms of indirect treatment comparisons but highlights a specific tool to assess the confidence of results of NMA. IQWIG does not recommend using indirect treatment comparisons routinely but describes specific contexts (e.g., new active substance within a medicinal product) for which NMA can play a role. NICE mentions that NMA may be additionally considered.

### Missing data

Regarding the NC on handling missing data in SRs, Cochrane highlights the importance of addressing it, summarizing a variety of approaches like sensitivity analysis. IQWIG uses a process of handling missing data encompassing a cut-off for the exclusion of a study.

### Bayesian methods

One NC addressed using Bayesian statistics in comparative effectiveness and safety assessments. According to Cochrane, Bayesian statistics may be problematic when combining objective trial data with subjective opinion but is strong in complex analyses like network meta-analyses. IQWIG suggests Bayesian methods can be used in SRs.

### Multiplicity

One NC addressed guidance on handling multiplicity (e.g., multiple outcomes/timepoints/effect measures). Cochrane stresses the importance of handling multiplicity in reviews, especially through developed pre-defined strategies in a review protocol. IQWIG uses adjustment methods for multiple testing where appropriate. No formal methods for adjustment for multiplicity are applied for the overall qualitative assessment of added benefit.

### Sensitivity analysis

One NC asks when sophisticated sensitivity analyses are deemed necessary within evidence syntheses and how these should be performed. Cochrane makes explicit suggestions (e.g., for missing data) for sensitivity analyses to test the robustness of overall findings. IQWIG and NICE also highlight the need for sensitivity analyses, describing various methods, especially for modeling approaches like health economic analyses.

## Reporting bias

One NC concerned to what extent reporting bias can be routinely considered within assessments. The need to consider reporting bias is highlighted by Cochrane. Methods such as inspection of missing studies and funnel plots are recommended. Formal scientific tools are mentioned and can be found in Supplementary Table D9. Cochrane further suggests considering potential conflicts of interest (COI) of study teams as an indication of sponsorship bias. SR authors should assess whether a COI is judged to represent a “notable concern” which can be included in the overview of studies tables. IQWIG also considers funnel plots, and unpublished data (such as CSR) are further highlighted to assess reporting bias fully.

## Certainty assessment

### Value judgments

NCs for this topic concern how (un)certainty in the evidence can be captured and to what extent this can be done without subjective value judgments, given EUnetHTA’s “partial use of GRADE.” Cochrane employs GRADE fully, noting that certainty of evidence is always the result of judgment and that GRADE ensures transparency of judgment. While IQWIG does not formally use GRADE, they state that benefit assessments are based on international standards of evidence-based medicine, such as those developed by the GRADE working group.

### Formulating recommendations

The NC in this topic asks whether and how to formulate recommendations. Cochrane advises against specific healthcare recommendations, encouraging authors to conclude on the evidence only. Conversely, NICE Committees formulate recommendations based on evidence of potential patient and healthcare system benefits.

## Other topics

### Patient and stakeholder involvement

Two NCs address stakeholder involvement methods and conflict of interest (COI) management. Cochrane describes several methods for involving patients and stakeholders such as surveys, workshops, focus groups, or participation in advisory groups. The decision about which method to use is based on available resources, and the review team should be aware of the likely benefits and possible limitations. Adequate support (training, funding) is needed. IQWIG involves stakeholders at the beginning of each project to define patient-relevant outcomes and subgroups. NICE also involves various experts in their assessments. Regarding COI, Cochrane requires declaration, with clear rules when authorship is not possible due to a potential COI. Stakeholders can become authors. IQWIG and NICE state that involved patients and other stakeholders need to declare their potential COI during nomination.

## Topics with NCs without any options in methodological handbooks

For nine topics, the authors did not identify any methodological solutions to included NCs. Some of these topics are related to procedural aspects relevant to the implementation of the HTAR (PICO survey, specifics on national adaptation of JCA). Supplementary Appendix G provides a list highlighting the availability of methodological solutions for specific NCs.

## Discussion

To the best of our knowledge, this is the first analysis to identify needs for concretization (NCs) in EUnetHTA guidance documents, rate their importance, and provide methodological options to address them. For some NCs, a single preferred approach could be identified among three internationally established methods guidebooks (primarily Cochrane). For others, the guidebooks offer vague recommendations, multiple options without preference, or no solution.

Methodological solutions were identified for numerous topics considered important for the work of AIHTA. These mainly concerned eligibility criteria (defining comparators, large number of RCTs), information sources (databases and additional sources), risk of bias (tools and handling studies with high RoB), synthesis methods (subgroup analysis, indirect treatment comparisons, and missing data), reporting bias (incl. Sponsorship bias), certainty assessment (value judgments, formulating a recommendation), and others (patient and stakeholder involvement).

In Austria, it is still undecided whether a supplementary document will be created to guide HTA authors in adapting JCAs or producing HTA reports using EUnetHTA guidelines under the HTAR. Our analysis may help HTA institutes develop or refine their methods, generally or for JCA adaptation. While some NCs can be resolved within overarching additional guidance, others will require experienced SR authors to decide case-by-case. This analysis may also support SR authors facing difficulties in applying EUnetHTA guidelines in specific contexts.

One clear strength of HTAR-mandated JCAs is that unpublished data from CSR will be available and incorporated. The related NC was only rated as somewhat important by the group discussion, likely linked to the fact that AIHTA generally excludes unpublished material. However, this change will likely play a pivotal role in addressing misreporting and other problems stemming from relying exclusively on published data (14–17). Harms, for instance, are often incomplete or under-reported in publications compared to unpublished data (18). Yet, using such data in SRs is highly complex, not least due to their quantity (14). The Restoring Invisible and Abandoned Trials (RIAT) initiative could help familiarize SR authors with the potential use of unpublished data (19). Methodological guidelines could reflect and define related best practices; Cochrane highlights this as a current guidance gap (10).

The main strength of this work is the comprehensive analysis identifying methodological solutions for identified NCs in numerous topics relevant to the work of HTA specialists. Yet, methodological considerations relevant to specific NCs were sometimes not described in full detail in the corresponding chapters in the guidebooks but addressed in sections with a different focus, or using a different terminology which is a common problem in the methodological literature (20). Despite our best efforts, certain details may have been overlooked. What is more, supplementary documents may offer further guidance not captured in the manuals analyzed here.

Furthermore, we only considered the guidebooks of three leading organizations. Fifty-four methodological documents (21) of HTA agencies in Europe and guidance from the GRADE working group in the form of published journal articles are available but were not considered. This was a pragmatic decision to reduce complexity and increase feasibility; it also reflected the wish to focus on regularly updated guidance documents to avoid potentially outdated guidance from published journal articles. However, even in the included documents, some recommendations were potentially outdated. For instance, all guidebooks recommended to use a checklist for subgroup analysis from 2010 (22;23) although the same group has published an update (the Instrument for Assessing the Credibility of Effect Modification Analyses, ICEMAN) (24;25).

For some needs, different solutions are available. For instance, for network meta-analyses, GRADE recommends the “minimally contextualized framework” (26), whereas the confidence in network-meta-analysis (CINEMA) approach (27) is recommended by Cochrane (10). EUnetHTA, IQWIG, and NICE do not mention scientific tools in this context; HTA institutes must decide which approach to adopt.

We noted a difference between HTA-oriented guidebooks (IQWIG and NICE) and SR-oriented guidelines (Cochrane). IQWIG and NICE tended to discuss options more vaguely, anticipating the need for adapting them to assessment-specific contexts and the potential consequences of adaptations. Conversely, the Cochrane Handbook provided more authoritative, mandatory guidance for Cochrane review authors, often without addressing how deviations might affect findings.

Some of the identified NCs related to both methodological and procedural guidance, particularly for national adaptation of JCAs. Our focus on methods documents naturally limited insights into procedural aspects. Finally, the prioritization of topics by AIHTA experts may not be generalizable to other HTA institutes.

Although our study is the first of its kind aiming to identify the need for more concrete guidance in EUnetHTA documents based on the perspective of HTA experts of one HTA institute, there are further analyses contrasting approaches of methodological and procedural challenges in HTA more broadly. For instance, the Adelaide Health Technology Assessment (AHTA) examined clinical evaluation methods in HTA, such as handling different types of evidence, further aspects such as consumer evidence, and broader topics, such as integrating equity into decision-making (28). AHTA conducted a scoping review (28) including 142 documents from twenty-two HTA agencies, with some overlaps of topics for which methodological approaches of HTA agencies were described. Our study differs from AHTA’s in scope and focus. We examined specific SR author needs with the perspective of one HTA institute as the starting point, while AHTA explored broader topics across multiple agencies using predefined themes from an Australian committee.

The HTACG methodology subgroup (6) meets regularly and updates some of the EUnetHTA guidelines analyzed in this study. As the HTAR does not prescribe a specific methodology, our synopsis of different methodological options may be helpful for both HTA bodies and the HTACG. Indeed, selected identified methodological NCs from this work have been submitted to the subgroup for consideration in updates. While the methodological guidance underpinning JCAs is intended to produce reports that can be used for decision-making in different healthcare systems and must therefore retain a degree of flexibility, this does not mean important issues should be left to ad hoc decisions. The challenge is striking a sensible balance between too concrete guidance that risks becoming dogmatic and overly situation-specific recommendations that can lead to highly heterogeneous approaches based on the same data.

EUnetHTA guidance recommends eliminating value judgments in JCAs, particularly when assessing the certainty of evidence. Consequently, the actual assessment is likely to occur at the national level during JCA adaptation, introducing new roles for authors of JCAs and those who will assess the technology’s added value. Which value judgments will be fully integrated in JCAs and which need to occur during national adaptation (e.g., RoB assessment) remains to be decided.

## Conclusion

Our analysis identified a need for concretization in some EUnetHTA guidelines. The structured overview of options based on methodological handbooks may support HTA doers in adapting and applying the guidelines to the national and local practical context. Synthesis methods, risk of bias, and certainty assessment are domains that could be prioritized, based on ranking and the availability of multiple methodological solutions.

## Supporting information

Goetz et al. supplementary material Goetz et al. supplementary material

## Supplementary material

The supplementary material for this article can be found at http://doi.org/10.1017/S0266462324004793.

## References

[1] European Commission EUR-Lex. Regulation (EU) 2021/2282 of the European Parliament and of the Council of 15 December 2021 on health technology assessment and amending Directive 2011/24/EU (Text with EEA relevance). [cited 01 May 2023]. Available from: http://data.europa.eu/eli/reg/2021/2282/oj.

[2] Imaz-Iglesia I , Wild C EUnetHTA’s contribution to the new legal framework for health technology assessment cooperation in Europe. Int J Technol Assess Health Care. 2022;38(1):e50. 35652661 10.1017/S026646232200037X

[3] Kristensen FB Mapping of HTA methodologies in EU and Norway. 2017 [cited 20 May 2023]. Available from: https://health.ec.europa.eu/system/files/2018-01/2018_mapping_methodologies_en_0.pdf.

[4] European Network for Health Technology Assessment (EUnetHTA). HTA Core Model®. Available from: https://www.eunethta.eu/hta-core-model/.

[5] European Network for Health Technology Assessment (EUnetHTA). Joint HTA Work. Available from: https://www.eunethta.eu/jointhtawork/.

[6] European Commission. Member State Coordination Group on HTA (HTACG). [cited 15 June 2023]. Available from: https://health.ec.europa.eu/health-technology-assessment/regulation-health-technology-assessment/member-state-coordination-group-hta-htacg_en.

[7] Austrian Institute for Health Technology Assessment (AIHTA). Methods. [cited 05 February 2024]. Available from: https://aihta.at/page/methoden/de.

[8] Page MJ , McKenzie JE , Bossuyt PM , Boutron I , Hoffmann TC , Mulrow CD , et al. The PRISMA 2020 statement: an updated guideline for reporting systematic reviews. BMJ. 2021;372:n71. 33782057 10.1136/bmj.n71 PMC8005924

[9] Harb SI , Tao L , Peláez S , Boruff J , Rice DB , Shrier I Methodological options of the nominal group technique for survey item elicitation in health research: a scoping review. J Clin Epidemiol. 2021;139:140–148. 34400255 10.1016/j.jclinepi.2021.08.008

[10] Higgins J , Thomas J , Chandler J , Cumpston M , Li T , Page M , et al. Cochrane Handbook for Systematic Reviews of Interventions. 2023. Available from: https://training.cochrane.org/handbook/current. 10.1002/14651858.ED000142 PMC10284251 31643080

[11] Institut für Qualität und Wirtschaftlichkeit im Gesundheitswesen (IQWIG). Allgemeine Methoden. Entwurf für Version 7.0. 2023 [cited 01 September 2023]. Available from: https://www.iqwig.de/methoden/allgemeine-methoden_version-7-0.pdf.

[12] National Institute for Health and Care Excellence (NICE). NICE health technology evaluations: the manual. Process and methods. 2022 [cited 15 June 2023]. Available from: https://www.nice.org.uk/process/pmg36/resources/nice-health-technology-evaluations-the-manual-pdf-72286779244741.

[13] Guyatt GH , Oxman AD , Kunz R , Woodcock J , Brozek J , Helfand M , et al. GRADE guidelines: 8. Rating the quality of evidence--indirectness. J Clin Epidemiol. 2011;64(12):1303–1310. 21802903 10.1016/j.jclinepi.2011.04.014

[14] Mahtani KR , Jefferson T , Heneghan C , Nunan D , Aronson JK What is a ‘complex systematic review’? Criteria, definition, and examples. BMJ Evid Based Med. 2018;23(4):127–130. 10.1136/bmjebm-2018-110965 29778991

[15] Jefferson T , Jørgensen L Redefining the ‘E’ in EBM. BMJ Evid Based Med. 2018;23(2):46–47. 10.1136/bmjebm-2018-110918 29595127

[16] Jefferson T Sponsorship bias in clinical trials: growing menace or dawning realisation? J R Soc Med. 2020;113(4):148–157. 32286115 10.1177/0141076820914242 PMC7160793

[17] Doshi P , Jefferson T Clinical study reports of randomised controlled trials: an exploratory review of previously confidential industry reports. BMJ Open. 2013;3(2):e002496. 10.1136/bmjopen-2012-002496 PMC3586134 23447465

[18] Hodkinson A , Gamble C , Smith CT Reporting of harms outcomes: a comparison of journal publications with unpublished clinical study reports of orlistat trials. Trials. 2016;17(1):207. 27103582 10.1186/s13063-016-1327-z PMC4840982

[19] Restoring Invisible & Abondoned Trials (RIAT). Glossary. [cited 18 April 2024]. Available from: https://restoringtrials.org/glossary/.

[20] Hirt J , Ewald H , Briel M , Schandelmaier S Searching a methods topic: practical challenges and implications for search design. J Clin Epidemiol. 2024;166:111201. 37914105 10.1016/j.jclinepi.2023.10.017

[21] Fuchs S , Olberg B , Panteli D , Busse R Health technology assessment of medical devices in europe: processes, practices, and methods. Int J Technol Assess Health Care. 2016;32(4):246–255. 27670589 10.1017/S0266462316000349

[22] Sun X , Briel M , Busse JW , You JJ , Akl EA , Mejza F , et al. Credibility of claims of subgroup effects in randomised controlled trials: systematic review. BMJ. 2012;344:e1553. 22422832 10.1136/bmj.e1553

[23] Sun X , Briel M , Walter SD , Guyatt GH Is a subgroup effect believable? Updating criteria to evaluate the credibility of subgroup analyses. BMJ. 2010;340:c117. 20354011 10.1136/bmj.c117

[24] Guyatt G , Zhao Y , Mayer M , Briel M , Mustafa R , Izcovich A , et al. GRADE guidance 36: updates to GRADE’s approach to addressing inconsistency. J Clin Epidemiol. 2023;158:70–83. 36898507 10.1016/j.jclinepi.2023.03.003

[25] Schandelmaier S , Briel M , Varadhan R , Schmid CH , Devasenapathy N , Hayward RA , et al. Development of the Instrument to assess the Credibility of Effect Modification Analyses (ICEMAN) in randomized controlled trials and meta-analyses. CMAJ. 2020;192(32):E901–e906. 32778601 10.1503/cmaj.200077 PMC7829020

[26] Brignardello-Petersen R , Florez ID , Izcovich A , Santesso N , Hazlewood G , Alhazanni W , et al. GRADE approach to drawing conclusions from a network meta-analysis using a minimally contextualised framework. BMJ 2020;371:m3900. 33177059 10.1136/bmj.m3900

[27] Nikolakopoulou A , Higgins JPT , Papakonstantinou T , Chaimani A , Del Giovane C , Egger M , et al. CINeMA: An approach for assessing confidence in the results of a network meta-analysis. PLoS Med. 2020;17(4):e1003082. 32243458 10.1371/journal.pmed.1003082 PMC7122720

[28] Adelaide Health Technology Assessment (AHTA). Paper 4: Clinical Evaluation Methods in HTA. [cited 07 October 2024]. Available from: https://www.health.gov.au/sites/default/files/2024-07/hta-policy-and-methods-review-hta-pathways-and-processes-clinical-evaluation-methods-and-horizon-scanning_0.pdf.

