# Supplementary material for: Implementation of the EU’s Health Technology Assessment regulation: where does existing methods guidance require concretization and what are the relevant methodological options?
Source: Int J Technol Assess Health Care. 2025 Feb 6;41(1):e9. doi: 10.1017/S0266462324004793 (PMC11811951; doi:10.1017/S0266462324004793)
Supplement: Goetz et al. supplementary material [file S0266462324004793sup001.docx]

Contents Appendix

[A: Code tree of qualitative analysis of guidance documents 3](#_Toc167893842)

[B: Team priority rating 4](#_Toc167893843)

[C: Needs for concretisation 7](#_Toc167893844)

[Table C1 EUnetHTA recommendations and text passages in need for concretisation: **eligibility criteria** 7](#_Toc167893845)

[Table C2: EUnetHTA recommendations and text passages in need for concretisation: **information sources** 8](#_Toc167893846)

[Table C3: EUnetHTA recommendations and text passages in need for concretisation: **search strategy** 9](#_Toc167893847)

[Table C4: EUnetHTA recommendations and text passages in need for concretisation: **data collection process** – no identified need for concretisation 10](#_Toc167893848)

[Table C5: EUnetHTA recommendations and text passages in need for concretisation: **data items** 11](#_Toc167893849)

[Table C6: EUnetHTA recommendations and text passages in need for concretisation: **risk of bias** 12](#_Toc167893850)

[Table C7: EUnetHTA recommendations and text passages in need for concretisation: **effect measures** 13](#_Toc167893851)

[Table C8: EUnetHTA recommendations and text passages in need for concretisation: **synthesis methods** 14](#_Toc167893852)

[Table C9: EUnetHTA recommendations and text passages in need for concretisation: **reporting bias** 15](#_Toc167893853)

[Table 10: EUnetHTA recommendations and text passages in need for concretisation: **certainty assessment** 16](#_Toc167893854)

[Table 11: Other identified topics and needs for concretisation 17](#_Toc167893855)

[D: EUnetHTA guidance documents eligible for screening/ group discussion 18](#_Toc167893856)

[E: Extraction Tables of methodological handbooks 20](#_Toc167893857)

[Table E1: Eligibility Criteria 20](#_Toc167893858)

[Table E2 Information sources 23](#_Toc167893859)

[Table E3: Search Strategy 24](#_Toc167893860)

[Table E4: Data collection – no needs for concretisation were identified 25](#_Toc167893861)

[Table E5: data items 26](#_Toc167893862)

[Table E6 Risk of Bias 27](#_Toc167893863)

[Table E7 effect measures 28](#_Toc167893864)

[Table E8 Synthesis methods 29](#_Toc167893865)

[Table D9: Reporting Bias 32](#_Toc167893866)

[Table E10: Certainty assessment 33](#_Toc167893867)

[Table D11: Other 34](#_Toc167893868)

[F: Documents used for analysis of methods handbooks 36](#_Toc167893869)

[Table F1: Handbooks and respective sources of handbooks 36](#_Toc167893870)

[Table F2: Core methods, specific perspective in review and other topics of Cochrane handbook 36](#_Toc167893871)

[G: Overview of rating whether methodological options represent methodological solutions on the basis of needs for concretisation 38](#_Toc167893872)

[H: Protocol: HTAR (HTA-Regulation) Implementation in Austria: Revision of the existing methods manual and complementation with national decision support methods 43](#_Toc167893873)

# A: Code tree of qualitative analysis of guidance documents


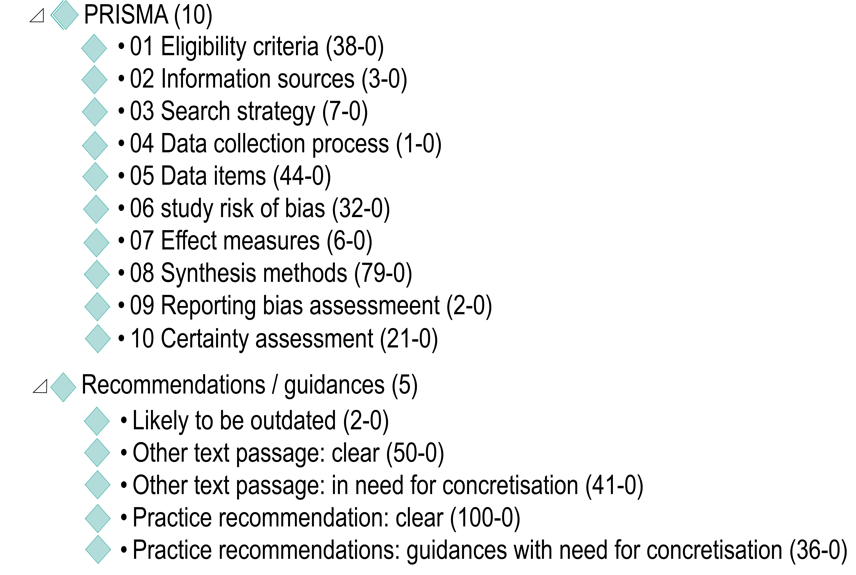


Abbreviations: PRISMA…Preferred Reporting Items for Systematic Reviews and Meta-Analyses

Numbering (01-10) refers to the predefined PRISMA domains. Numbers in brackets refer to the number of coded text passages.

# B: Team priority rating

Table B1: Overview of needs for concretisation and team priority rating

| PRISMA domains | Reference | Topic | Need | Source | Mean | Median | Min | Max | n of respondents |
| --- | --- | --- | --- | --- | --- | --- | --- | --- | --- |
| 01 Eligibility criteria | 1\| 1 | Logic models | Use of logic models in scoping phase | Screening | 2,20 | 2 | 1 | 4 | 10 |
|  | 1\| 2 | Defining the intervention | Product-specific | Screening | 3,60 | 3,5 | 1 | 6 | 10 |
|  | 1\| 3 | Eligibility of NRSI | Comparative effectiveness | Screening | 5,00 | 5 | 4 | 6 | 10 |
|  | 1\| 4 | Other incident reporting databases | Incident reporting databases | Screening | 3,50 | 3,5 | 2 | 5 | 10 |
|  | 1\| 5 | Eligibility of NRSI | Retrospective studies | Screening | 4,30 | 5 | 1 | 6 | 10 |
|  | 1\| 6 | Eligibility of NRSI | Single-arm studies | Screening | 5,10 | 5 | 4 | 6 | 11 |
|  | 1\| 7 | Unpublished data | Clinical study reports (CSR) | Screening | 3,36 | 3 | 2 | 6 | 11 |
|  | 1\| 8 | PICO survey - key competencies | Key competencies | Screening | 4,44 | 4 | 2 | 6 | 9 |
|  | 1\| 9 | PICO survey - role of MS | Role of MS | Screening | 4,83 | 5 | 3 | 6 | 6 |
|  | 1\| 10 | Indication change | CE-mark change | Screening | 3,25 | 4 | 1 | 5 | 8 |
|  | 1\| 11 | Comparator | Defining Comparator | Screening | 4,82 | 5 | 4 | 6 | 11 |
|  | 1\|12 | Large number of RCTs | valid cut-off? | Team | 4,00 | 4 | 4 | 4 | 1 |
| 02 information sources | 2\|1 | Databases | Setting a minimum of databases | Team | 5,50 | 5,5 | 5 | 6 | 4 |
|  | 2\|2 | Additional sources | Selection further sources | Team | 5,00 | 5 | 4 | 6 | 4 |
| 03 Search strategy | 3\|1 | Search strategy | peer review | Screening | 2,75 | 2,5 | 1 | 5 | 8 |
| 04 Data collection process | NA | - | - | - | - | - | - | - | - |
| 05 Data items | 5\|1 | Composite endpoints | disaggregating safety endpoints | Screening | 4,43 | 4 | 3 | 6 | 7 |
|  | 5\|2 | Safety outcome reporting | use of MeDRA | Screening | 3,86 | 4 | 3 | 5 | 7 |
| 06 Risk of bias | 5\|1 | RoB Tools | Endpoint RoB v.2 | Screening | 5,13 | 5 | 4 | 6 | 8 |
|  | 5\|2 | RoB Tools | ROBINS-I: study vs. endpoint level | Screening | 5,13 | 5 | 4 | 6 | 8 |
|  | 5\|3 | RoB Tools | Numerous tools listed | Screening | 5,13 | 5 | 4 | 6 | 8 |
|  | 5\|4 | RoB Tools | RoB of uncontrolled trials (e.g., case series) | Screening | 5,25 | 5 | 5 | 6 | 8 |
|  | 5\|5 | RoB Tools | Quality of safety data | Screening | 4,00 | 4 | 3 | 5 | 6 |
|  | 5\|6 | Registry and RWD | Study design vs. data | Screening | 4,88 | 5 | 3 | 6 | 8 |
|  | 5\|7 | Studies with high RoB | Dealing with high RoB | Screening | 4,38 | 4,5 | 3 | 6 | 8 |
| 07 Effect measures | 7\|1 | Context factors | Incorporating context factors and user dependency in evidence synhtesis | Screening | 2,83 | 3 | 1 | 4 |  |
| 08 Synthesis methods | 8\| 1 | Applicability | Role of MS | Screening | 3,30 | 3 | 3 | 4 | 10 |
|  | 8\| 2 | Applicability | Re-analysis | Screening | 2,88 | 3 | 1 | 4 | 8 |
|  | 8\| 3 | Subgroup analysis | How and when to consider potential effect modification | Screening | 4,00 | 4 | 3 | 5 | 9 |
|  | 8\| 4 | Subgroup analysis | How to assess credibility of subgroup analysis | Screening | 4,33 | 4 | 3 | 5 | 9 |
|  | 8\| 5 | Indirect treatment comparison | How and when to conduct network meta-analysis | Screening | 3,33 | 3 | 2 | 4 | 9 |
|  | 8\| 6 | Indirect treatment comparison | How to synthesise evidence from NMA | Screening | 3,78 | 4 | 3 | 4 | 9 |
|  | 8\| 7 | Indirect treatment comparison | When and how indirect comparisons should be undertaken. | Screening | 4,00 | 4 | 3 | 5 | 9 |
|  | 8\| 8 | Indirect treatment comparison | How evidence derived from indirect treatment comparisons should be adequately assessed/ synthesised | Screening | 4,33 | 4 | 3 | 5 | 9 |
|  | 8\| 9 | Causal modelling methods | Propensity-score | Screening | 4,29 | 4 | 4 | 5 | 7 |
|  | 8\| 10 | Missing data | Missing data: addressing impact on synthesis | Screening | 4,11 | 4 | 2 | 6 | 9 |
|  | 8\| 11 | Statistical methods | Bayesian approach: when to consider in addition to standard hypothesis testing | Screening | 3,25 | 3,5 | 1 | 5 | 8 |
|  | 8\| 12 | Multiplicity | Addressing problem of multiplicity | Screening | 4,13 | 4,5 | 2 | 6 | 8 |
|  | 8\| 13 | Sensitivity analysis | When sophisticated sensitivity analysis deemed to be necessary | Screening | 3,50 | 3 | 2 | 5 | 10 |
|  | 8\| 14 | Post-hoc analysis | EUnetHTA suggests reporting of post-hoc analysis according to EMA. No standardised way at AIHTA. | Screening | 3,90 | 3,5 | 3 | 5 | 10 |
|  | 8\| 15 | Post-hoc analysis | Considering post-hoc analysis (incl. weight of such analysis within synthesis) | Screening | 4,50 | 4,5 | 3 | 6 | 10 |
| 09 Reporting bias | 9\|1 | Reporting bias, incl. Sponsorship bias | Considering reporting bias in published trials | Team | - | - | - | - |  |
| 10 Certainty assessment | 10\|1 | Value judgements | Using GRADE without certain GRADE terminology (EUnetHTA's "negative list" of terminology to be avoided) | Screening | 4,40 | 5 | 3 | 5 | 5 |
|  | 10\|2 | Value judgements | Using GRADE certainty of evidence context-independently | Screening | 4,33 | 4,5 | 3 | 5 | 6 |
|  | 10\|3 | Value judgements | EUnetHTA recommends GRADE judgements to be "non-judgemental" | Screening | 3,83 | 4,5 | 1 | 5 | 6 |
|  | 10\|4 | Value judgements | EUnetHTA recommends formulating a conclusion without value judgements | Screening | 3,71 | 4 | 1 | 5 | 7 |
|  | 10\|5 | Formulating a recommendation | Whether and how should SR authors formulate recommendations | Screening | - | - | - | - | 1 |
|  | 10\|6 | National adaptation | It is unclear how a national adaptation can take place | Screening | 4,29 | 4 | 3 | 6 | 7 |
| 11 Other | 11\|1 | Patient and stakeholder involvement | Clarify how patients and other stakeholders should be involved | Team | - | - | - | - | 1 |
|  | 11\|2 | Patient and stakeholder involvement | How should a potential COI be identified/ handled? | Team | - | - | - | - | 1 |

*Abbreviations:* AIHTA…Austrian Institute for Health Technology Assessment; CE…conformité européenne; COI…conflict of interest; CSR…clinical study reports; EMA… European Medicines Agency; EUnetHTA… European Network for Health Technology Assessment; GRADE… Grading of Recommendations Assessment, Development and Evaluation; Max…maximum; MedDRA… Medical dictionary for regulatory activities; Min…minimum; MS…member state; n…number; NMA…network meta-analysis; NRSI…non-randomised studies of interventions; PICO…population, intervention, control and outcomes; RCT…randomised controlled trial; RoB…risk of bias; RWD… real-world data; SR…systematic review;

Figure B1: Importance rating: closed response rating

Importance rating: How important is the issue for your work practice?

6 … Very important

5 … Important

4 … Somewhat important

3 … Somewhat unimportant

2 … Unimportant

1 … Not important at all

x … I don’t understand the statement

# C: Needs for concretisation

### Table C1 EUnetHTA recommendations and text passages in need for concretisation: **eligibility criteria**

| Topic | EUnetHTA (keywords) | Need for concretisation |
| --- | --- | --- |
| Use of logic models in scoping phase | Logic model, analytical framework | EUnetHTA suggests that logic models (e.g., analytical frameworks) may be used during scoping. These are currently not used at AIHTA. |
| Defining the intervention | Assessment of a MD product vs. assessment of intervention (STA, MTA) | At AIHTA, product-specific assessments are not conducted, whereas EUnetHTA also considers evaluation of particular products in the assessment scope. |
| Eligibility of NRSI and other databases | Comparative effectiveness | It is unclear which specific NRSI are eligible to be included in comparative effectiveness assessments. |
|  | Retrospective studies | AIHTA often excluded retrospective studies by default, which is in slight contrast to EUnetHTA. |
|  | Single-Arm Studies | It is unclear, in how far single-arm studies should be included |
| Other incident databases | Incident reporting databases for safety analysis | It is unclear in how far non-published databases should be included in systematic reviews (e.g., incident reporting databases). |
| Eligibility of unpublished data | Clinical study reports | EUnetHTA recommends including unpublished data such as clinical study reports (CSR). It is unclear, how far CSR should be incorporated and which methods should be applied in doing so. |
| PICO survey | Role of member states | Member states should provide information required for defining PICO questions. It is unclear who has key competencies to inform PICO question and in how far these can be extrapolated to a whole country. |
|  |  | It is unclear how and who should define a “national” policy question”. |
| Indication change | CE mark change | It is unclear, in how far a team should react towards a change within indications during the review process. |
| Comparator | Defining comparators | EUnetHTA recommends using the “more economic therapy” as the comparator. It is unclear which criteria should be used to select most appropriate comparators. |
| Large number of RCTs | Large number of RCTs eligibility | Is there anything on valid cut off methods to reduce the amount of evidence to a feasible amount? |

Abbreviations: AIHTA…Austrian Institute for Health Technology Assessment; CE…conformité européenne; CSR…clinical study reports; EUnetHTA… European Network for Health Technology Assessment; GRADE… Grading of Recommendations Assessment, Development and Evaluation; MD…medical device; MTA…multi-technology assessment; NRSI…non-randomised studies of interventions; PICO…population, intervention, control and outcomes; RCT…randomised controlled trial; STA… single technology assessment.

### Table C2: EUnetHTA recommendations and text passages in need for concretisation: **information sources**

| Topic | EUnetHTA (keywords) | Need for concretisation |
| --- | --- | --- |
| Databases |  | Setting a minimum of requirement of databases routinely searched – (quasi-) independent of the topic/research question (e.g. according to the Cochrane Handbook of SRs), both for literature (e.g. Medline) and data on clinical Trials (e.g. ClinicalTrials.gov) |
| Additional sources |  | Selection of (additional) information sources/databases searched – depending on the topic/research question |

Abbreviations: EUnetHTA…European Network for Health Technology Assessment; SR…systematic review.

### Table C3: EUnetHTA recommendations and text passages in need for concretisation: **search strategy**

| Topic | EUnetHTA (keywords) | Need for concretisation |
| --- | --- | --- |
| Search strategy | Peer Review of search strategy | There is currently no peer-review process of the search strategy, being recommended by EUnetHTA.  (apart from the recommendations in **EUnetHTA guideline JA3 2016-2020**, § 3.1.6 and 3.2.5)  *Time permitting, a peer review of the search strategy should (ideally) be conducted → Level: “recommended” not “mandatory” |

Abbreviations: EUnetHTA… European Network for Health Technology Assessment.

### Table C4: EUnetHTA recommendations and text passages in need for concretisation: **data collection process** – no identified need for concretisation

### Table C5: EUnetHTA recommendations and text passages in need for concretisation: **data items**

| Topic | EUnetHTA (keywords) | Need for concretisation |
| --- | --- | --- |
| Composite outcomes | Disaggregation recommended | It is unclear how to disaggregate composite safety endpoints for reporting. |
| Safety outcome reporting | MedDRA Dictionary | At AIHTA, MedDRA is currently not used for describing adverse events being recommended by EUnetHTA. |

*Abbreviations:* AIHTA…Austrian Institute for Health Technology Assessment; EUnetHTA… European Network for Health Technology Assessment; MedDRA… Medical dictionary for regulatory activities;

### Table C6: EUnetHTA recommendations and text passages in need for concretisation: **risk of bias**

| Topic | EUnetHTA (keywords) | Need for concretisation |
| --- | --- | --- |
| RoB Tools | Study vs. endpoint level | Cochrane RoB v.2^[[1]](#footnote-1)^ is currently often visualised on a study-level at AIHTA. EUnetHTA recommends using each tool on an endpoint level. |
|  | Study vs. endpoint level | ROBINS-I^[[2]](#footnote-2)^ is currently often visualised on a study-level at AIHTA. whereas EUnetHTA recommends using each tool on an endpoint level. |
|  | Numerous tools listed | It is unclear which (key) tools should or should not be used according to EUnetHTA for specific study designs. |
|  | RoB of uncontrolled studies (single-arm studies, cross-sectional studies, case series, etc.) | EUnetHTA suggests that no RoB tool needs to be used as these studies are inherently biased, while AIHTA usually uses the IHE-20 checklist^[[3]](#footnote-3)^. |
|  | Quality of safety data | EUnetHTA recommends to “(…) evaluate risk of bias of both sources of information and quality of data on adverse reaction”. Numerous questions are listed helping to guide authors, without clear guidance on how to actually assess the “data on adverse reactions”. |
| Registry and RWD | Study design vs. data | EUnetHTA suggests that Registries and RWD are not study designs per se and hence should not be considered as studies. At AIHTA, there is no uniform way how to consider registries and studies derived thereof. It is unclear in this context how systematic review authors should account for double counting of patients if numerous studies use the same database (e.g. a registry). |
| Studies with high RoB | Dealing with high RoB | EUnetHTA broadly defines four options for dealing with a high or unclear risk of bias (excluding studies with high RoB, perform sensitivity analysis, describing uncertainty, combination of the two latter options) |

Abbreviations: AIHTA…Austrian Institute for Health Technology Assessment; EUnetHTA… European Network for Health Technology Assessment; RoB…risk of bias; RWD… real-world data.

### Table C7: EUnetHTA recommendations and text passages in need for concretisation: **effect measures**

| Topic | EUnetHTA (keywords) | Need for concretisation |
| --- | --- | --- |
| Context factors | User dependency and context factors | While considering factors such as user dependency and other context factors is recommended by EUnetHTA, it is unclear how to incorporate these factors rigorously within evidence synthesis. |

Abbreviation: EUnetHTA… European Network for Health Technology Assessment.

### Table C8: EUnetHTA recommendations and text passages in need for concretisation: **synthesis methods**

| Topic | EUnetHTA (keywords) | Need for concretisation |
| --- | --- | --- |
| Applicability | External validity | EUnetHTA suggests assessing applicability, but also that the final decision on applicability should be left to the discretion of each member state. It is unclear how to assess applicability rigorously within this approach. |
|  | External validity | EUnetHTA suggests that studies may be re-analysed if these are not applicable without specifying how such analysis can be performed. |
| Subgroup analysis | Subgroup effect, effect modification | EUnetHTA recommends addressing potential effect modification. It is unclear, when potential effect modification should be considered and how. |
|  | Subgroup effect, effect modification | EUnetHTA provides information on effect modification, but it is generally unclear how the credibility of a subgroup effect should be assessed and what should be specifically be reported. |
| Indirect treatment comparison | Network Meta-Analysis (NMA) | It is unclear if and when NMA should be conducted. |
|  | Network Meta-Analysis (NMA) | It is unclear how to synthesise evidence from/ assess credibility of existing NMA. |
|  | Indirect comparison | EUnetHTA describes different forms of indirect comparisons and an “initial feasibility question”. It remains unclear, when and how indirect comparisons should be undertaken. |
|  | Indirect comparisons | It is unclear how evidence derived from indirect treatment comparisons should be adequately assessed/ synthesised. |
| Causal modelling methods | Propensity Score | It is unclear how evidence derived from propensity-score matched comparisons should be adequately assessed for risk of bias/ synthesised. |
| Missing data | Impact of missing data | EUnetHTA recommends addressing the potential impact of missing data within the findings of evidence syntheses. It remains unclear, which specific approaches are feasible within reviews. |
| Bayesian statistical methods | Bayesian statistics | Bayesian statistics are described within EUnetHTA documents. It remains unclear how Bayesian in addition or instead of frequentist meta-analysis can be for assessing effectiveness and safety. |
| Multiplicity |  | The problem of multiplicity (e.g., multiple outcomes, multiple time points, multiple effect measures) is described within EUnetHTA documents incl. statistical methods that are availalble to correct for multiplicity. However, no clear guidance on how to address multiplicity adequately is provided. |
| Sensitivity analysis |  | EUnetHTA considers sensitivity analysis as important, although it remains unclear when sophisticated sensitivity analyses are deemed necessary or unnecessary within evidence syntheses and how these should be performed. |
| Post-hoc analysis |  | EUnetHTA suggests that reporting of post-hoc analyses should follow the principles outlined in the EMA guideline. At AIHTA, there is currently no standardised way of how post-hoc analyses are reported and left to the discretion of the systematic review author. |
|  |  | It is unclear to which extent post-hoc analyses should be considered within evidence syntheses and how much weight can be attributed to these analyses. |

Abbreviatons: AIHTA…Austrian Institute for Health Technology Assessment; EMA… European Medicines Agency; EUnetHTA… European Network for Health Technology Assessment; NMA…network meta-analysis.

### Table C9: EUnetHTA recommendations and text passages in need for concretisation: **reporting bias**

| Topic | EUnetHTA (keywords) | Need for concretisation |
| --- | --- | --- |
| Reporting bias (incl. sponsorship bias) |  | It is unclear how to identify and address potential reporting bias incl. sponsorship bias within clinical trials. |

*Abbreviation:* EUnetHTA… European Network for Health Technology Assessment.

### Table C10: EUnetHTA recommendations and text passages in need for concretisation: **certainty assessment**

| Topic | EUnetHTA (keywords) | Need for concretisation |
| --- | --- | --- |
| Value judgements | Partial use of GRADE | EUnetHTA created a “negative list” of phrases not to use within JCA as these incorporate value judgements. It is unclear how conclusions can be drawn on evidence without using any of these phrases and without any value judgements. |
|  | Partial use of GRADE | EUnetHTA considers using GRADE context-independently. It is unclear how certainty of evidence can be determined context-independently. |
|  | Partial use of GRADE | EUnetHTA recommends that “context-independent judgements can be formulated more conclusively than context-dependent issues”. Further it is recommended that These judgements should not be judgemental. It is unclear how a judgement can be formulated in a non-judgemental way. |
|  | Conclusion | EUnetHTA recommends formulating a conclusion without value judgements and suggest to concisely summarise the results. It is unclear if such conclusion can be written without being a summary only. |
| Formulating a recommendation | Formulating a recommendation | This need for concretisation refers to the question whether and how HTAs should formulate recommendations. |
| National adaptation | contextualisation | A joint clinical assessment is considered an intermediate product in need for contextualisation by member states. It is unclear, how a national adaptation can take place. |

Abbreviations: AIHTA…Austrian Institute for Health Technology Assessment; EUnetHTA… European Network for Health Technology Assessment; GRADE… Grading of Recommendations Assessment, Development and Evaluation; HTA…health technology assessment; JCA…joint clinical assessment.

### Table C11: Other identified topics and needs for concretisation

| Topic | EUnetHTA (keywords) | Need for concretisation |
| --- | --- | --- |
| Patient and stakeholder involvement | Involvement | Clarify how patients and other stakeholders should be involved |
| Patient and stakeholder involvement | COI | How should a potential COI be identified/ handled? |

Abbreviations: COI…conflict of interest; EUnetHTA…European Network for Health Technology Assessment.

# D: EUnetHTA guidance documents eligible for screening/ group discussion

Information retrieval missing?

| Document name | Version | Publication date:  year | URL |
| --- | --- | --- | --- |
| D4.6 VALIDITY OF CLINICAL STUDIES | 1.0 | 2022 | <https://www.eunethta.eu/wp-content/uploads/2022/12/EUnetHTA-21-D4.6-Practical-Guideline-on-validity-of-clinical-studies-v1.0-1.pdf> |
| Internal validity of randomised controlled trials | 2.0 | 2015 | <https://www.eunethta.eu/wp-content/uploads/2018/01/16_WP7-SG3-GL-int_val_RCTs_amend2015.pdf> |
| Applicability of evidence for the context of a relative effectiveness assessment | 2.0 | 2019 | <https://www.eunethta.eu/wp-content/uploads/2018/01/Levels-of-Evidence-Applicability-of-evidence-for-the-context-of-a-relative-effectiveness-assessment_Amended-JA1-Guideline_Final-Nov-2015.pdf> |
| Process of information retrieval for systematic reviews and health technology assessments on clinical effectiveness | 2.0 | 2019 | <https://www.eunethta.eu/wp-content/uploads/2020/01/EUnetHTA_Guideline_Information_Retrieval_v2-0.pdf> |
| Therapeutic medical devices | 1.0 | 2015 | <https://www.eunethta.eu/wp-content/uploads/2018/01/Therapeutic-medical-devices_Guideline_Final-Nov-2015.pdf> |
| D4.2 SCOPING PROCESS | 1.0 | 2022 | <https://www.eunethta.eu/wp-content/uploads/2022/09/EUnetHTA-21-D4.2-practical-guideline-on-scoping-process-v1.0.pdf> |
| D4.3.1: DIRECT AND INDIRECT COMPARISONS | 1.0 | 2022 | <https://www.eunethta.eu/wp-content/uploads/2022/12/EUnetHTA-21-D4.3.1-Direct-and-indirect-comparisons-v1.0.pdf> |
| D4.3.2  DIRECT AND INDIRECT COMPARISONS | 1.0 | 2022 | <https://www.eunethta.eu/wp-content/uploads/2022/08/EUnetHTA-21-Deliverable-D4.3.2-Methodological-Guideline-on-Direct-and-indirect-comparisons-V1.0.pdf> |
| D4.4 – OUTCOMES (ENDPOINTS) | 1.0 | 2023 | <https://www.eunethta.eu/wp-content/uploads/2023/01/EUnetHTA-21-D4.4-practical-guideline-on-Endpoints-v1.0.pdf> |
| D4.5 – APPLICABILITY OF EVIDENCE – PRACTICAL GUIDELINE ON  MULTIPLICITY, SUBGROUP, SENSITIVITY AND POST HOC ANALYSES | 1.0 | 2022 | <https://www.eunethta.eu/wp-content/uploads/2022/12/EUnetHTA21-D4.5-Practical-Guideline-on-Applicability-of-Evidence-v1.0.pdf> |
| COMPARATORS & COMPARISONS  Criteria for the choice of the most appropriate comparator(s)  Summary of current policies and best practice recommendations | 2.0 | 2015 | <https://www.eunethta.eu/wp-content/uploads/2018/01/WP7-SG3-GL-choice_of_comparator_amend2015.pdf> |
| COMPARATORS & COMPARISONS:  Direct and indirect comparisons | 2.0 | 2015 | <https://www.eunethta.eu/wp-content/uploads/2018/01/Comparators-Comparisons-Direct-and-indirect-comparisons_Amended-JA1-Guideline_Final-Nov-2015.pdf> |
| Endpoints used for Relative Effectiveness Assessment:  HEALTH-RELATED QUALITY OF LIFE and UTILITY MEASURES | 2.0 | 2015 | <https://www.eunethta.eu/wp-content/uploads/2018/01/Endpoints-used-for-Relative-Effectiveness-Assessment-Health-related-quality-of-life-and-utility-measures_Amended-JA1-Guideline_Final-Nov-2015.pdf> |
| Endpoints used for Relative Effectiveness Assessment:  Clinical Endpoints | 2.0 | 2015 | <https://www.eunethta.eu/wp-content/uploads/2018/02/WP7-SG3-GL-clin_endpoints_amend2015.pdf> |
| Endpoints used for Relative Effectiveness Assessment  Composite endpoints | 2.0 | 2015 | <https://www.eunethta.eu/wp-content/uploads/2018/01/Endpoints-used-for-Relative-Effectiveness-Assessment-Composite-endpoints_Amended-JA1-Guideline_Final-Nov-2015_0.pdf> |
| Endpoints used in Relative Effectiveness Assessment: Safety | 2.0 | 2015 | <https://www.eunethta.eu/wp-content/uploads/2018/03/WP7-SG3-GL-safety_amend2015.pdf> |
| Endpoints used in Relative Effectiveness Assessment: Surrogate Endpoints | 2.0 | 2015 | <https://www.eunethta.eu/wp-content/uploads/2018/03/surrogate_endpoints.pdf> |
| Internal validity of non-randomised studies (NRS) on interventions | 1.0 | 2015 | <https://www.eunethta.eu/wp-content/uploads/2018/01/Internal-validity-of-non-randomised-studies-NRS-on-interventions_Guideline_Final-Jul-2015.pdf> |
| ‘negative list’ Task Group for Common Phrases and GRADE | 1.0 | 2019 | <https://www.eunethta.eu/wp-content/uploads/2021/05/EUnetHTA-Negative-list-of-phrases.pdf> |
| Partial Use of GRADE in EUnetHTA Framework | 1.0 | 2020 | <https://www.eunethta.eu/wp-content/uploads/2021/05/EUnetHTA-GRADE-framework-paper.pdf> |
| EUnetHTA Common phrases – i.e. recommendations for formulations | 1.0 | 2019 | <https://www.eunethta.eu/wp-content/uploads/2021/05/EUnetHTA-Commom-Phrases.pdf> |
| JOINT CLINICAL ASSESSMENT OF HIGH-RISK MEDICAL DEVICES  D4.7.1 Synthesis of national requirements  D4.7.2 Framework for the assessment of high-risk medical devices and in vitro  diagnostics | 1.0 | 2022 | <https://www.eunethta.eu/wp-content/uploads/2022/07/EUnetHTA-21-Deliverable-D4.7.1-D4.7.2-General-Guidance-Framework-for-high-risk-MDs_V1.0.pdf> |

All documents were accessed in July, 2023

# E: Extraction Tables of methodological handbooks

### Table E1: Eligibility Criteria

| Topic | NCs | Cochrane | IQWIG | NICE |
| --- | --- | --- | --- | --- |
| Logic models | 1\|1 | Logic models are judged to be useful when intervention complexity is a key consideration. It can further be useful in systematic reviews when considering whether failure to find a beneficial effect of an intervention is due to a theory failure, an implementation failure or both. (see 2.5.1) | NR | NR |
| Defining the intervention | 1\|2 | Reviews are intervention based (not product based) | Reviews are intervention based (not product based) | NICE appraisals are based on an intervention or technology level. |
| Eligibility of NRSI and other databases | 1\|3- 1\|6 | Focus on RCTs. Valid justifications for inclusion of NRSI needed (see Ch. 24)   - RCTs not feasible, but RCT evidence address the research questions only indirectly or incompletely: E.g., rare outcomes, different study population or setting. - RCTs are not feasible (intervention of interest cannot or are very unlikely to be randomised (e.g., population-level interventions such as the effect and harm of a certain legislation or other interventions where high patient preferences (for a certain intervention) would prevent randomisation.   Further justifications/ reasons sometimes described for including NRSI in systematic reviews include if weaknesses of available NRSI are to be studies, if an intervention effect is very large, or if only a small number of RCTs are available. Cochrane states that caution is urged within these justifications as some of these (such as large effects justification) are implicitly result-driven/ post-hoc arguments and certain value-judgements (opinion) or evidence would be required to inform the argument. | Focus on RCTs. Exceptions can be made in contexts that may justify lower-level evidence, such as the presence of a dramatic effect (see Ch. 3.2.2)  IQWIG operationalizes the presence of a dramatic effect based on the signal-to-noise model^[[4]](#footnote-4)^. An effect is considered dramatic if it is significant at the level of 1% and expressed as a relative risk exceeding 10. This criterion is not a rigid threshold, but serves as an orientation. | Focus (“strong preference”) on RCTs.  NRSI are considered to be beneficial to substitute RCTs (if none are available) or supplement them in specific situations (i.e., to contextualise results by, for instance, assessing generalisability of results from RCTs, show effectiveness over a longer time horizon, provide data on experience of people; see 3.3.11). |
| Unpublished Data | 1\|7 | Cochrane recommends searching for unpublished data such as clinical study reports (CSR). Cochrane states that guidance on how to use data from regulatory sources is needed (Ch. 4).  Review authors should search for unpublished sources of data related to adverse events such as clinicaltrials.gov to identify safety data (see 19.3.4 Including unpublished sources). | IQWIG considers unpublished data through submission dossiers (3.2.1) | NICE considers all types of evidence in its evaluations incl. unpublished data. Manufacturers are asked to provide study reports (see 1.3.3. and 1.3.38)  NICE considers it to be important to identify relevant evidence that's not publicly available during evaluations, such as unpublished or incomplete clinical trial data and post-marketing surveillance data (see 3.3.25) |
| PICO survey | 1\|8, 1\|9 | NR | NR | NR |
| CE-Mark/ Indication change during review | 1\|10 | NR | NR | NR |
| Comparator | 1\|11 | NR | NR | The committee chooses suitable comparators by following the established practices of the NHS, the natural history of the medical condition, NICE guidance, cost-effectiveness, and regulatory status. Any practices that prove to be cost-ineffective after economic analysis may be excluded. Long-standing treatments that lack regulatory approval or unregulated use of technology can also be considered as comparators if they are part of clinical practice. However, safety and efficacy evidence is also taken into account. |
| Large number of RCTs | 1\|12 | Cochrane states that available RCTs should fundamentally be incuded if they match the review question. However, some exclusion criteria may be considered depending on the research question(s), for instance regarding (3.3.1 and MECIR 3.3.b):   - cluster or crossover RCTs - no use of placebo (for the comparator group), - evaluation of outcomes unblinded to allocation sequence - period of follow-up (e.g. short follow-up in studies investigating long-term conditions) | NR | NR |

*Abbreviations:* AIHTA…Austrian Institute for Health Technology Assessment; CE…conformité européenne; Ch…. chapter; CSR…clinical study reports; EUnetHTA… European Network for Health Technology Assessment; IQWIG…Institut für Qualität und Wirtschaftlichkeit im Gesundheitswesen; NHS…National Health Service; NICE… National Institute for Health and Care Excellence; NR…not reported; NRSI…non-randomised studies of interventions; PICO…population, intervention, control and outcomes; RCT…randomised controlled trial.

### Table E2 Information sources

| Topic | NCs | Cochrane | IQWIG | NICE |
| --- | --- | --- | --- | --- |
| Databases | 2\|1 | Cochrane states that general bibliographic databases (Medline, and Embase if access is availale) and CENTRAL within Cochrane reviews should be searched to identify published evidence (mandatory element). A search of MEDLINE alone is not considered adequate. Furthermore, trial registries and repositories through clinicaltrials.gov, WHO International Trials Registry Platform (ICTRP) portal and further sources should be searched as appropriate (Ch.4). | IQWIG mentions that it is required to search multiple bibliographic databases and mentions the guidance of Cochrane hereby (Ch.8.1.1)  For focused searches (e.g., used for rapid revies), at least two medical databases^[[5]](#footnote-5)^ should be used and adapted to the specific topic (Ch. 8.2.).  At least two Trial registries are used for the identification of ongoing studies: clinicaltrials.gov and WHO ICTRP | NR |
| Searching other Sources | 2\|2 | Cochrane considers it to be highly desirable to search for grey literature (reports, dissertations, conference abstracts) and within previous reviews. Checking the reference lists in included studies is further considered mandatory. Regulatory agency sources and clinical study reports are considered to be increasingly important sources (see 4.3.4 and 4.3.5). | IQWIG highlights that further sources may be used (e.g., sources of regulatory bodies, conference proceedings; 8.1.4) | To ensure that the evaluation does not miss important relevant evidence, it is  important that attempts are made to identify evidence that is not in the public  domain. Such evidence includes unpublished clinical trial data and clinical trial  data that are in abstract form only or are incomplete, and post-marketing  surveillance data. However, this evidence should still consider the key principles  of design, analysis and reporting (Ch. 3.3.25) |

Abbreviations: Ch…. chapter; IQWIG…Institut für Qualität und Wirtschaftlichkeit im Gesundheitswesen; NICE… National Institute for Health and Care Excellence; NR…not reported; WHO ICTRP…World Health Organisation International Clinical Trials Registry Platform.

### Table E3: Search Strategy

| Topic | NCs | Cochrane | IQWIG | NICE |
| --- | --- | --- | --- | --- |
| Search strategy peer review | 3\|1 | Cochrane strongly recommends that the search strategy is peer reviewed (Ch. 4.2.2) | NR | NR |

Abbreviations: Ch…. chapter; IQWIG…Institut für Qualität und Wirtschaftlichkeit im Gesundheitswesen; NICE… National Institute for Health and Care Excellence; NR…not reported.

### Table E4: Data collection – no needs for concretisation were identified

### Table E5: data items

| Topic | NCs | Cochrane | IQWIG | NICE |
| --- | --- | --- | --- | --- |
| Composite endpoints | 5\|1 | Cochrane states that composite measures may be drilled down for details of distinct adverse events, without clarifying precise methods in doing so if safety reporting is sparse (Ch. 19.1.2.1) | If sufficient data are available, sensitivity  analyses can be carried out with the exclusion versus addition of individual components.  Under appropriate conditions, individual endpoints can be determined and calculated from  endpoints from a combined endpoint can be determined and calculated (Ch. 9.1.5). | NR |
| Safety outcome reporting | 5\|2 | Any coding system or standard medical terminology such as COSTART or MedDRA are considered useful pieces of information for safety reporting. | IQWIG considers MedDRA to be an important standardised coding system. | NR |

*Abbreviations:* Ch…. chapter; COSTART… Coding Symbols for a Thesaurus of Adverse Reaction Terms; IQWIG…Institut für Qualität und Wirtschaftlichkeit im Gesundheitswesen; MedDRA…Medical Dictionary for Regulatory Activities; NICE… National Institute for Health and Care Excellence; NR…not reported;

### Table E6 Risk of Bias

| Topic | NCs | Cochrane | IQWIG | NICE |
| --- | --- | --- | --- | --- |
| RoB Tools | 5\|1-5\|5 | RCTs: Cochrane suggests assessing RoB on endpoint level for both RCTs (see Ch. 8) and NRSI (see Ch.25), with the Cochrane RoB v.2 and ROBINS-I tool highlighted as the preferred tools.  There is no statement or recommended approach specifically for lower-level studies such as single-arm trials.  Cochrane recommends to use the same tools also for assessing RoB of safety data. Further tools that specifically address RoB of safety data not pre-specified in the study protocol are mentioned (e.g., McHarm tool, AHRQ assessment tool) | IQWIG use their own RoB tools not fully openly accessible and assess the RoB both on endpoint-level and study-level (see section 9.1.4). These have similar domains to the tools recommended by Cochrane.  Specific RoB tools of single-arm studies or of safety data are not reported. | NICE mentions the use of validated tools (such as ROBINS-I), without clearly mentioning which tools to use for which study design.  Specific RoB tools for single-arm studies or of safety data are not reported. |
| Registry and RWD  (double counting) | 5\|6 | NR | NR | NR |
| Studies with high RoB | 5\|7 | Fundamentally, RCTs should be included in the review. Some inclusion criteria related to quality (e.g., use of placebo or follow-up time period, blinding to allocation sequence) may be defined a priori. Cochrane recognises the need to balance between restricting inclusion criteria to minimise bias and the number of studies likely to fulfil these criteria (Ch. 3.3.1).  Cochrane recommends an overall risk of bias assessment for a result of a study without an overall risk of bias judgment of a study following the latest risk of bias tool (Cochrane RoB v.2; Ch. 8). | NR | NR |

*Abbreviations:* AHRQ… Agency for Healthcare Research and Quality; Ch…. chapter; IQWIG…Institut für Qualität und Wirtschaftlichkeit im Gesundheitswesen; NICE… National Institute for Health and Care Excellence; NR…not reported; RoB…risk of bias; RWD… real-world data.

### Table E7 effect measures

| Topic | NCs | Cochrane | IQWIG | NICE |
| --- | --- | --- | --- | --- |
| Context factors | 7\|1 | Cochrane suggests that intervention complexity may also be reflected within “simple” intervention if analysed in their wider context. For conceptualising context factors, these simple interventions may be regarded as complex, with the following questions being asked. First, it should be reflected how the intervention itself may be complex. Second, one may reflect in which specific situation implementation may result in (complex) interactions. Third, the focus of analysis may shift from an individual intervention to a wider context within the intervention is implemented (Ch. 17).  However, Cochrane suggests that review authors should be aware that there is always some variation, making it impossible to investigate on all conceivable differences. Hence, it may be sufficient to consider context factors using the logic model and further identified subgroups for synthesis (Ch. 17) | NR | NR |

*Abbreviations:* Ch…. chapter; IQWIG…Institut für Qualität und Wirtschaftlichkeit im Gesundheitswesen; NICE… National Institute for Health and Care Excellence; NR…not reported;

### Table E8 Synthesis methods

| Topic | NCs | Cochrane | IQWIG | NICE |
| --- | --- | --- | --- | --- |
| Applicability | 8\|1, 8\|2 | Cochrane suggests addressing applicability within the discussion and conclusion of the review authors findings. The GRADE approach is hereby mentioned (see Ch. 15). | IQWIG mentions importance of applicability assessment, but does not specify a specific method. | NR |
| Subgroup analysis | 8\|3, 8\|4 | Subgroup analyses can be performed on subsets of participants or studies, such as males and females or different geographic locations. They help investigate heterogeneous results and answer specific questions about patient groups, interventions, or study types. (see Ch. 10.11.2).  It is necessary to employ a formal statistical test to compare different subgroups. Simply relying on differences in the level of statistical significance within subgroups to conclude that there is a difference in effect can be highly misleading. (Ch. 10)  Checklist by Sun et al. ^[[6]](#footnote-6)^ is referenced (Ch. 15). | Detailed description of limitations of subgroup analysis (Ch. 9.3.9).  Checklist by Sun et al. 6 is referenced. | In considering subgroup analyses,re met. The need for proper documentation (e.g., the evidence supporting biological/ clinical plausibility) is highlighted and the checklist by Sun et al.6 is referenced. |
| Indirect treatment comparison | 8\|5-8\|8 | There are no decision rules when NMA should be undertaken.  Cochrane mentions the CINEMA tool^[[7]](#footnote-7)^ within the context of available NMA | IQWIG does not recommend to utilise methods of indirect treatment comparisons routinely, while highlighting the role for indirect treatment comparisons in specicic contexts, e.g., new active substances in medicinal products (Ch.9.3.8) | NICE considers NMA additionally if appropriate. It should be presented as such. |
| Causal modelling methods | 8\|9 | NR | NR | NR |
| Missing data | 8\|10 | Cochrane highlights potential sources of missing data: whole studies, certain outcomes or summary data of an outcome may be missing (Ch. 10.12).  Four ways to handle missing data are listed:   1. Analyzing only available data. 2. Imputing missing data with replacement values and treating them as observed. 3. Imputing missing data while accounting for the imputed uncertainty. 4. Using statistical models that take into account the relationship between the missing data and the available data while making certain assumptions. (Ch. 10.12.2)   Cochrane states general recommendations to deal with missing data:   1. Contact original investigators for missing data 2. Explicitly state assumptions of methods used 3. Follow guidance in Chapter 8 to assess risk of bias 4. Perform sensitivity analyses to assess results' sensitivity to assumptions 5. Address the impact of missing data in the discussion section. (see Ch. 10.12.2) | Missing data are evaluated in a 2-step process. First, participants who were completely excluded from the analyses are considered. Results are generally not included in the benefit assessment if they are based on less than 70% of the study participants. Second, the potential for confounding due to follow-up losses is considered, and no fixed limits are set for the assessment. The number, time points, and reasons for follow-up losses increase the potential for bias. If the number of missing values is too high or unsuitable replacement strategies were used, the results may not be taken into account. (Ch. 9.3.11) | NR |
| Bayesian statistics | 8\|11 | Cochrane states that Bayesian statistics is based on a different philosophy. Cochrane highlights that many regard it to be controversial to combine objective trial data with subjective opinion, which is (often) the case if Bayesian meta-analyses use a weakly or non-informative prior distributions. However, several strengths are equally listed; e.g., when performing complex analyses such as NMA or when examining the extent to which the available data would change the beliefs of the people (see Ch. 10). | IQWIG states that Bayesian methods (as an alternative to classic statistical testing) can be considered within systematic reviews; e.g., in meta analyses or for indirect comparisons (Ch. 9.3.2, 9.3.7 and 9.3.8) | NR |
| Multiplicity | 8\|12 | Cochrane regards it to be necessary to specify in the review protocol how authors will handle the multiplicity of outcomes outcomes (Chapter 3). This should address the following: multiplicity due to multiple outcomes, including but not limited to multiple outcomes measured within a domain (e.g., depression), multiple methods for measuring an outcome (e.g., self-reported vs. clinician-rated depression) or when multiple time points are measured within a time frame. Further, multiplicity may also arise from with regard to reporting of multiple analyses of the same outcome (e.g., different analyses such as intention-to treat and per-protocol analyses).  *Cochrane further states that it may be difficult for SR authors to anticipate all forms of multiplicity within the scoping phase of a review/ the review protocol. Therefore, authors should note all post-hoc approaches to select both outcomes or results within the methods section (Ch. 3).* | IQWIG states that the problem of multiple testing cannot be formally solved within systematic reviews more broadly, but should be taken into account. Where appropriate and possible, IQWIG applies adjustment methods for multiple testing. For benefit assessments (see Section 3.1), the Institute endeavours to assess the type 1 error separately for each individual benefit aspect by, for example, using the cross survival time data, for example, the cross-time analysis is primarily used. The  summarising assessment across all outcomes is not usually carried out in a quantitative manner, so that no formal methods of adjustment for multiple testing are used here (Section 9.3.2 and Ch. 3.1) | NR |
| Sensitivity analysis | 8\|13 | Sensitivity analyses should be used to examine whether overall findings are robust to potentially influential decisions (Ch. 10) | IQWIG considers it to be necessary to conduct both univariate, multivariate and deterministic sensitivity analyses within health economic modelling (e.g., 4.11).  Further, sensitivity analyses may be undertakten within benefit assessments, e.g., between higher ranked outcomes with regard to benefits and harms of an intervention (3.1.1) | NICE mentions clear examples when sensitivity analyses should be undertaken including but not limited to: doubt about relevance of a study within am meta-analysis (3.4.10, 3.4.13), selecting prior distributions within NMA (3.4.18), when measuring valuing health effects in cost-utility analyses (4.3.8, 3.3.9) for evidence on resource use and costs (4.4.5, 4.4.10, 4.4.21), Non-NHS and non-PSS costs (4.4.24) when there are plausible alternative assumptions within modelling methods (4.6.1., 4.6.11, 4.6.27) for addressing uncertainty (4.7.6, 4.7.7., 4.7.8) |
| Post-hoc analysis | 8\|14, 8\|15 | NR | NR | NR |

*Abbreviations*: Ch…. chapter; GRADE… Grading of Recommendations Assessment, Development and Evaluation; IQWIG…Institut für Qualität und Wirtschaftlichkeit im Gesundheitswesen; NICE… National Institute for Health and Care Excellence; NMA…network meta-analysis; NR…not reported; SR…systematic review.

### Table E9: Reporting Bias

| Topic | NCs | Cochrane | IQWIG | NICE |
| --- | --- | --- | --- | --- |
| Reporting bias (incl. sponsorship bias) | 9\|1 | Cochrane highlights the importance of a reporting bias assessment: “(…) any methods used to assess the risk of bias due to missing results should be described. Such methods may include consideration of the number of studies missing from a synthesis due to selective non-reporting of results, or investigations to assess small-study effects (e.g. funnel plots), which can arise from the suppression of small studies with ‘negative’ results (also called publication bias). If relevant, any tools or checklists used (such as ROB-ME^[[8]](#footnote-8)^) should be cited (Ch. 3 and Ch. 13). See Chapter 13 for a description of methods for assessing risk of bias due to missing results in a synthesis.  With regard to sponsorship bias, Cochrane considers it to be highly desirable to address conflict of interests in included studies: “(…) Review authors should consider assessing whether they judge a trial to be of ‘notable concern about conflicts of interest’. This assessment is useful for exploration of possible heterogeneity between trials (e.g. in a subgroup analysis), and for reflection on relevant mechanisms for how conflict of interest may have biased trial results and synthesis results. Concerns about conflicts of interest can be reported in the ‘Characteristics of included studies’ table..” (Ch. 7) | IQWIG specifically highlights the need for including unpublished data such as clinical study reports (CSR) to fully assess reporting bias (3.2.1).  IQWIG searches for and considers material from unpublished sources (8.1).  IQWIG considers funnel plots for assessing the likelihood of publication bias (9.3.13). | NR |

*Abbreviations*: Ch…. chapter; CSR…clinical study reports; IQWIG…Institut für Qualität und Wirtschaftlichkeit im Gesundheitswesen; NICE… National Institute for Health and Care Excellence; NR…not reported.

### Table E10: Certainty assessment

| Topic | NCs | Cochrane | IQWIG | NICE |
| --- | --- | --- | --- | --- |
| Value Judgements | 10\|1-10\|4 | Cochrane uses GRADE and highlights that the certainty of evidence is “(….) a result of judgement, but the judgement process operates within a transparent structure” (Ch. 14.1.6.8)  It is expected within a Cochrane review to use the five GRADE considerations within a Cochrane report (MECIR Box 14.2.a).  With regard to conclusions, Cochrane suggests narrative statements to draw a conclusion of the evidence incorporating the effect estimate and the certainty of evidence. For each GRADE certainty of evidence (high, moderate, low, very low) there are one to four suggested statements, for which the intervention (X) and comparator (Y) of the specific Cochrane report are to be inserted (Ch.15). | IQWIG does not use GRADE formally, but highlights that benefit assessments and the (un)certainty in the results are based on international standards such as those developed by the GRADE working group (see 3.1.4). | NR |
| Formulating a recommnendation | 10\|5 | According to Cochrane, it is not the role of review authors to provide recommendations on healthcare decisions. They should focus on describing the certainty of evidence, the balance of benefits and harms, as well as highlight various actions that are consistent with different values, preferences, cost, and other factors that influence decision-making (Ch. 15) | NR | NICE committees formulate one of the following recommendations based on whether the patients, health and social care system potentially benefits from the intervention: recommended, recommended in specific circumstances, recommended with managed access, recommended with data collection, recommended only in a research context, or not recommended |
| National adaptation |  | NR | NR | NR |

*Abbreviations:* Ch…. chapter; GRADE… Grading of Recommendations Assessment, Development and Evaluation; IQWIG…Institut für Qualität und Wirtschaftlichkeit im Gesundheitswesen; NICE… National Institute for Health and Care Excellence; NR…not reported.

### Table E11: Other

| Topic | NCs | Cochrane | IQWIG | NICE |
| --- | --- | --- | --- | --- |
| Patient and stakeholder involvement | 11\|1 | “The involvement of consumers and other stakeholders can be helpful in ensuring that the categories of data collected are sufficiently aligned with the needs of review users” (Ch. 1, section 1.3)  “Methods for working with consumers and other stakeholders include surveys, workshops, focus groups and involvement in advisory groups. Decisions about what methods to use will typically be based on resource availability, but review teams should be aware of the merits and limitations of such methods. Authors will need to decide who to involve and how to provide adequate support for their involvement. This can include financial reimbursement, the provision of training, and stating clearly expectations of involvement, possibly in the form of terms of reference.” | IQWIG regards the involvement of stakeholders as an established international standard. Stakeholder involvement primarily occurs at the beginning of each assessment in the context of the definition of patient-relevant outcomes and relevant subgroups. For reports and rapid reports, the involvement takes place as an oral (e.g., reports or rapid reports), written (e.g., dossier assessments) or no (e.g., assessment according to § 137h SGB) consultation with patients takes place. An additional user test and experience reports is further considered for the website health information (“gesundheitsinformation.de”).  Ch. 2, Section 2.2.1 | “Clinical experts, patient experts and commissioning experts provide their  views and experience throughout the evaluation. They help clarify issues  that the technical team has identified, give written evidence, participate  in any technical engagement (when needed), and attend the committee  meeting (if required) Section 1.3.8 |
|  | 11\|2 | Cochrane clarifies COI to be declared for all authors, with clear rules when authorship is not possible due do potential COI. Stakeholders / consumers can become authors (Ch.1.3) | IQWIG considers it to be necessary to declare potential conflicts of interests via a form “form the disclosure of relationships” (Ch. 2.2.3) | Any organisation and expert (clinical, patient, etc.) must complete a nomination form that includes a conflicts of interest disclosusre (Ch. 1.3.16) |

*Abbreviations:* Ch…. chapter; COI…conflict of interest; IQWIG…Institut für Qualität und Wirtschaftlichkeit im Gesundheitswesen; NICE… National Institute for Health and Care Excellence; NR…not reported.

# F: Documents used for analysis of methods handbooks

### Table F1: Handbooks and respective sources of handbooks

| Organisation, year | Version | Link | Assessed |
| --- | --- | --- | --- |
| IQWIG | 7.0 | <https://www.iqwig.de/methoden/allgemeine-methoden_version-7-0.pdf> | 01.10.2023 |
| NICE | NA | <https://www.nice.org.uk/process/pmg36/resources/nice-health-technology-evaluations-the-manual-pdf-72286779244741> | 01.10.2023 |
| Cochrane | V.6.4 | <https://training.cochrane.org/handbook/current> | 01.10.2023 |

Abbreviations: IQWIG…Institut für Qualität und Wirtschaftlichkeit im Gesundheitswesen; NICE… National Institute for Health and Care Excellence.

### Table F2: Core methods, specific perspective in review and other topics of Cochrane handbook

| Title | Chapter |
| --- | --- |
| [Starting a review](https://training.cochrane.org/handbook/current/chapter-01) | 1 |
| [Determining the scope and questions](https://training.cochrane.org/handbook/current/chapter-02) | 2 |
| [Inclusion criteria & grouping for synthesis](https://training.cochrane.org/handbook/current/chapter-03) | 3 |
| [Searching & selecting studies](https://training.cochrane.org/handbook/current/chapter-04) | 4 |
| [Collecting data](https://training.cochrane.org/handbook/current/chapter-05) | 5 |
| [Effect measures](https://training.cochrane.org/handbook/current/chapter-06) | 6 |
| [Bias and conflicts of interest](https://training.cochrane.org/handbook/current/chapter-07) | 7 |
| [Risk of bias in randomized trials](https://training.cochrane.org/handbook/current/chapter-08) | 8 |
| [Preparing for synthesis](https://training.cochrane.org/handbook/current/chapter-09) | 9 |
| [Meta-analyses](https://training.cochrane.org/handbook/current/chapter-10) | 10 |
| [Network meta-analyses](https://training.cochrane.org/handbook/current/chapter-11) | 11 |
| [Synthesis using other methods](https://training.cochrane.org/handbook/current/chapter-12) | 12 |
| [Bias due to missing results](https://training.cochrane.org/handbook/current/chapter-13) | 13 |
| [‘Summary of findings’ tables & GRADE](https://training.cochrane.org/handbook/current/chapter-14) | 14 |
| [Interpreting results](https://training.cochrane.org/handbook/current/chapter-15) | 15 |
| [Equity](https://training.cochrane.org/handbook/current/chapter-16) | 16 |
| [Intervention complexity](https://training.cochrane.org/handbook/current/chapter-17) | 17 |
| [Patient-reported outcomes](https://training.cochrane.org/handbook/current/chapter-18) | 18 |
| [Adverse effects](https://training.cochrane.org/handbook/current/chapter-19) | 19 |
| [Economic evidence](https://training.cochrane.org/handbook/current/chapter-20) | 20 |
| [Qualitative evidence](https://training.cochrane.org/handbook/current/chapter-21) | 21 |
| [Prospective approaches](https://training.cochrane.org/handbook/current/chapter-22) | 22 |
| [Variants on randomized trials](https://training.cochrane.org/handbook/current/chapter-23) | 23 |
| [Including non-randomized studies](https://training.cochrane.org/handbook/current/chapter-24) | 24 |
| [Risk of bias in non-randomized studies](https://training.cochrane.org/handbook/current/chapter-25) | 25 |
| [Individual participant data](https://training.cochrane.org/handbook/current/chapter-26) | 26 |

# G: Overview of rating whether methodological options represent methodological solutions on the basis of needs for concretisation

| **PRISMA** | **Reference ID** | **Topic** | **NC** | **Source** | **Importance Mean** | **Importance Median** | **Methodological solution** | | |
| --- | --- | --- | --- | --- | --- | --- | --- | --- | --- |
|  |  |  |  |  |  |  | **Cochrane** | **IQWIG** | **NICE Consensus** |
|  | 1\|1 | Logic models | Use of logic models in scoping phase |  | 2,20 | 2 | somew hat | No | No |
|  | 1\| 2 | Defining the intervention | Product-specific | Scre ening | 3,60 | 3,50 | Yes | Yes | somew hat |
|  | 1\| 3 | Eligibility of NRSI | Comparative effectiveness | Screening | 5,00 | 5,00 | somewhat | somewhat | somewhat |
|  | 1\| 4 | Other incident databases | Incident Reporting databases | Screening | 3,50 | 3,50 | No | No | No |
|  | 1\| 5 | Eligibility of NRSI | Retrospective studies | Screening | 4,30 | 5,00 | No | No | No |
|  | 1\| 6 | Eligibility of NRSI | Single-arm studies | Screening | 5,10 | 5,00 | No | No | No |
|  | 1\| 7 | Unpublished data | CSR | Screening | 3,36 | 3,00 | somewhat | somewhat | somewhat |
|  | 1\| 8 | PICO survey - key competencies | Key competencies | Screening | 4,44 | 4,00 | No | No | No |
|  | 1\| 9 | PICO survey - role of MS | Role of MS | Screening | 4,83 | 5,00 | No | No | No |
|  | 1\| 10 | Indication change | CE mark change | Screening | 3,25 | 4,00 | No | No | No |
|  | 1\| 11 | Comparator | Defining Comparator | Screening | 4,82 | 5,00 | No | No | Yes |
|  | 1\|12 | Large number of RCTs | valid cut-off? | Team | 4,00 | 4,00 | Yes | No | No |
| 02 information sources | 2\|1 | Databases | Setting a minimum of databases | Team | - | - | Yes | Yes | No |
|  | 2\|2 | Additional sources | Selection further sources | Team | - | - | Yes | Yes | Yes |
| 03 Search strategy | 3\|1 | Search strategy | peer review | Screening | 2,75 | 2,50 | Yes | No | No |
| 04 Data collection process | | |  |  |  |  |  |  |  |
| 05 Data items | 5\|1 | Composite endpoints | disaggregating safety endpoints | Screening | 4,43 | 4,00 | No | No | No |
|  | 5\|2 | Safety outcome reporting | use of MeDRA | Screening | 3,86 | 4,00 | Yes | Yes | No |
| 06 Risk of bias | 6\|1 | RoB Tools | Endpoint RoB v.2 | Screening | 5,13 | 5,00 | Yes | Yes | somewhat |
|  | 6\|2 | RoB Tools | ROBINS-I: study vs. endpoint level | Screening | 5,13 | 5,00 | Yes | Yes | somewhat |
|  | 6\|3 | RoB Tools | Numerous tools listed | Screening | 5,13 | 5,00 | No | No | No |
|  | 6\|4 | RoB Tools | RoB of uncontrolled trials (e.g., case series) | Screening | 5,25 | 5,00 | No | No | No |
|  | 6\|5 | RoB Tools | Quality of safety data | Screening | 4,00 | 4,00 | Yes | No | No |
|  | 6\|6 | Registry and RWD | Study design vs. data | Screening | 4,88 | 5,00 | No | No | No |
|  | 6\|7 | Studies with high RoB | Dealing with high RoB | Screening | 4,38 | 4,50 | Yes | No | No |
| 07 Effect measures | 7\|1 | Context factors | Incorporating context factors and user dependency in evidence synhtesis | Screening | 2,83 | 3,00 | No | No | No |
| 08 Synthesis methods | 8\| 1 | Applicability | Role of Member state | Screening | 3,30 | 3,00 | Yes | No | No |
|  | 8\| 2 | Applicability | Re-analysis | Screening | 2,88 | 3,00 | No | No | No |
|  | 8\| 3 | Subgroup analysis | How and when to consider potential effect modification | Screening | 4,00 | 4,00 | somewhat | somewhat | somewhat |
|  | 8\| 4 | Subgroup analysis | How to assess credibility of subgroup analysis | Screening | 4,33 | 4,00 | Yes | Yes | Yes |
|  | 8\| 5 | Indirect treatment comparison | How and when to conduct network meta-analysis | Screening | 3,33 | 3,00 | somewhat | somewhat | somewhat |
|  | 8\| 6 | Indirect treatment comparison | How to synthesise evidence from NMA | Screening | 3,78 | 4,00 | Yes | No | No |
|  | 8\| 7 | Indirect treatment comparison | When and how indirect comparisons should be undertaken. | Screening | 4,00 | 4,00 | No | No | No |
|  | 8\| 8 | Indirect treatment comparison | How evidence derived from indirect treatment comparisons should be adequately assessed/ synthesise | Screening | 4,33 | 4,00 | No | No | No |
|  | 8\| 9 | Causal modelling | Propensity-score | Screening | 4,29 | 4,00 | No | No | No |
|  | 8\| 10 | Missing data | Missing data: addressing impact on synthesis | Screening | 4,11 | 4,00 | Yes | Yes | No |
|  | 8\| 11 | Statistical methods | Bayesian approach: when to consider in addition to standard hypothesis testing | Screening | 3,25 | 3,50 | Yes | Yes | No |
|  | 8\| 12 | Multiplicity | Addressing problem of multiplicity | Screening | 4,13 | 4,50 | somewhat | somewhat | No |
|  | 8\| 13 | Sensitivity analysis | When sophisticated sensitivity analysis deemed to be necessary | Screening | 3,50 | 3,00 | Yes | Yes | Yes |
|  | 8\| 14 | Post-hoc analysis | EUnetHTA suggests reporting of post-hoc analysis according to EMA. No standardised way at AIHTA. | Screening | 3,90 | 3,50 | No | No | No |
|  | 8\| 15 | Post-hoc analysis | Considering post-hoc analysis (incl. weight of such analysis within synthesis) | Screening | 4,50 | 4,50 | No | No | No |
| 09 Reporting bias | 9\|1 | Reporting bias, incl. Sponsorship bias | Considering reporting bias in published trials | Team | - | - | Yes | somewhat | No |
| 10 Certainty assessment | 10\|1 | Partial use of GRADE | Using GRADE without certain GRADE terminology (EUnetHTA's "negative list" of terminology to be avoided) | Screening | 4,40 | 5,00 | Yes | somewhat | No |
|  | 10\|2 | Partial use of GRADE | Using GRADE certainty of evidence context-independently | Screening | 4,33 | 4,50 | Yes | somewhat | No |
|  | 10\|3 | Partial use of GRADE | EUnetHTA recommends GRADE judgements to be "non-judgemental" | Screening | 3,83 | 4,50 | Yes | somewhat | No |
|  | 10\|4 | Conclusion | EUnetHTA recommends formulating a conclusion without value judgements | Screening | 3,71 | 4,00 | Yes | somewhat | No |
|  | 10\|5 | Formulating a recommendation | Whether and how should SR authors formulate recommendations | Screening | - | - | Yes | No | Yes |
|  | 10\|6 | National adaptation | It is unclear how a national adaptation can take place | Screening | 4,29 | 4,00 | No | No | No |
| 11 Other | 11\|1 | Patient and stakeholder involvement | Clarify how patients and other stakeholders should be involved | Team | - | - | Yes | Yes | Yes |
|  | 11\|2 | Patient and stakeholder involvement | COI | Team | - | - | Somewhat | Yes | Yes |

*Abbreviations:* AIHTA…Austrian Institute for Health Technology Assessment; CE…conformité européenne; COI…conflict of interest; CSR…clinical study reports; EMA… European Medicines Agency; GRADE… Grading of Recommendations Assessment, Development and Evaluation; MedDRA… Medical dictionary for regulatory activities; MS…member state; NMA…network meta-analysis; NRSI…non-randomised studies of interventions; PICO…population, intervention, control and outcomes; RCT…randomised controlled trial; RoB…risk of bias; RWD… real-world data; SR…systematic review.

# H: Protocol: HTAR (HTA-Regulation) Implementation in Austria: Revision of the existing methods manual and complementation with national decision support methods


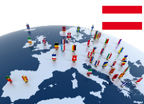


**Research area:** HTA-methods & steering instruments

**Duration:** June 2023 – April 2024
**Language:** English (with German summary)

**Background:**
In January 2022, the EU regulation on the EU-wide joint assessment of health technologies (Health Technology Assessment Regulation, HTAR) was adopted [1]. It will be legally binding in January 2025 after a transition period of three years [2]. Under the HTAR, cooperation in the evaluation, especially of medicinal products and medical devices, are regulated. It may provide a structure for carrying out joint HTA using state-of-the-art methods on the European level by producing methodologically rigorous and timely scientific assessments supporting evidence-based decisions on the national level [2].

In most EU member states, the national institutionalisation of HTA increasingly took place within the past two decades. The European Network for Health Technology Assessment (EUnetHTA) built the basis for scientific and technical cooperation between HTA bodies [3]. After establishing the EUnetHTA project (2006-2009), further activities, primarily in subsequent joint actions, contributed to building and upholding HTA capacity and trust [3]. Due to a high degree of overlap between the HTA methodology of EUnetHTA rapid relative effectiveness (REA) and assessments of HTA bodies [3], the use of these joint assessments at a national level ("national uptake") was feasible and reduced redundancies.

Within the latest project (EUnetHTA21), further deliverables in the form of, inter alia, extensive updates of existing guidance and new guidance for joint assessments were created to facilitate the implementation of the HTAR [4]. Currently, the Member State Coordination Group on HTA (HTACG) builds on the aforementioned deliverables from EUnetHTA and established subgroups aiming at developing both methodological and procedural guidance for HTAR. The HTACG also aims to ensure cooperation between EU bodies such as the European Medicines Agency (EMA), other Medical Device Coordination Group (MDCG) expert panels and the appropriate involvement of stakeholder organisations and experts in its work [5].

Although the core methodological guidance is generally well established within the EUnetHTA Core Model® [3, 4], some of the recommendations from EUnetHTA guidances leave room to decide between alternative methodological options.

**Project Objective and Research Questions:**
This project aims to support a national adaptation of EUnetHTA guidelines by identifying these EUnetHTA guidances with need for concretisation, describing alternative methodological options based on different methods manuals and supporting a decision on the most appropriate option for the AIHTA, with specific focus on assessments for the Austrian hospital benefit catalogue and the individual medical services (MEL) .

The following research questions (RQ) will, hence, be answered:

- **RQ1:** Which EUnetHTA guidances provide room to decide among alternative methodological options or are in need for concretisation?
- **RQ2:** How can these alternative methodological options described based on different methodological handbooks?
- **RQ3:** Which of these alternative methodological options are most suitable for Austrian rapid assessments supporting a decision on the annual revision of the hospital benefit catalogue?

**Methods:**
**Identification of EUnetHTA guidances with room to decide among alternative methodological options**: EUnetHTA guidelines will be systematically reviewed based on sections and sub-sections. One or more of the following criteria must apply to be considered a guideline recommendation with need for concretisation:

- Broadly defined guidance stating different options instead of giving clear guidance.
- The divergence between EUnetHTA guidelines and AIHTA methods paper or standard practice at AIHTA.
- General unclarity concerning what is proposed within a EUnetHTA guidance.

**Synopsis of different alternative methodological options based on methodological handbooks**: The following organisation's manuals are reviewed with a specific focus on the preferred alternative methodological option of the organisation. The following organisations/ documents will hereby be selected:

- IQWIG/ IQWIG methods handbook 6.0 [6].
- NICE/ HTA manual [7].
- Cochrane/ Cochrane Handbook [8].

Further, a focused search in the LIGHTS database [9]will be conducted to identify other methods handbooks. In this context, any official methodological guidance documents (e.g., manuals, handbooks) by European HTA organisations and EBM institutions are eligible.

**Defining the most appropriate option among alternative approaches:** After identifying guidances with need for concretisation and preferred alternative methodological option of different organisations, the results will be presented to experts from Austrian HTA organisations and the AIHTA team members. Depending on their feedback and willingness to cooperate within a broader methods manual, either a external review phase or a semi-structured discussion ideally using a voting system (e.g., DELPHI method) may then be used to decide on the most appropriate alternative methodological option to be used for the AIHTA reflecting both expert input from other HTA organisations and the lived practice of AIHTA systematic review authors.

**Timeplan:**

| **Time period** | **Deliverable** |
| --- | --- |
| June, July 2023 | Scoping and drafting protocol, identification of guidances with need for concretisation |
| August, September 2023 | Synopsis of different alternative methodological options |
| October 2023 | Drafting the report, presenting preliminary results to Austrian HTA organisation and coordination of methods paper |
| November 2023 | Drafting preliminary version, optional: DELPHI method for Austrian concretisation |
| December 2023- February 2024 | Peer-review |
| March – April 2024 | Publication of revised AIHTA report/ methods manual |

**References**:

[1]  European Commission EUR-Lex. Regulation (EU) 2021/2282 of the European Parliament and of the Council of 15 December 2021 on health technology assessment and amending Directive 2011/24/EU (Text with EEA relevance). [cited 01.05.2023]. Available from: <http://data.europa.eu/eli/reg/2021/2282/oj>.

[2]  I. Imaz-Iglesia and C. Wild. EUnetHTA's contribution to the new legal framework for health technology assessment cooperation in Europe. Int J Technol Assess Health Care. 2022;38(1):e50. Epub 20220602. DOI: 10.1017/s026646232200037x.

[3]  F. B. Kristensen. Mapping of HTA methodologies in EU and Norway. 2017 [cited 20.05.2023]. Available from: <https://health.ec.europa.eu/system/files/2018-01/2018_mapping_methodologies_en_0.pdf>.

[4]  European Network for Health Technology Assessment (EUnetHTA). Joint HTA Work. Available from: <https://www.eunethta.eu/jointhtawork/>.

[5] European Commission. Member State Coordination Group on HTA (HTACG). [cited 15.06.2023]. Available from: <https://health.ec.europa.eu/health-technology-assessment/regulation-health-technology-assessment/member-state-coordination-group-hta-htacg_en>.

[6]  Institut für Qualität und Wirtschaftlichkeit im Gesundheitswesen (IQWIG). Allgemeine Methoden. Entwurf für Version 7.0. 2022 [cited 01.05.2023]. Available from: <https://www.iqwig.de/methoden/allgemeine-methoden_entwurf-fuer-version-7.pdf>.

[7] National Institute for Health and Care Excellence (NICE). NICE health technology evaluations: the manual. Process and methods 2022 [cited 15.06.2023]. Available from: <https://www.nice.org.uk/process/pmg36/resources/nice-health-technology-evaluations-the-manual-pdf-72286779244741>.

[8]  J. Higgins, J. Thomas, J. Chandler, M. Cumpston, T. Li, M. Page, et al. Cochrane Handbook for Systematic Reviews of Interventions. Cochrane Handbook for Systematic Reviews of Interventions. 2019. DOI: 10.1002/9781119536604.

[9]  J. Hirt, C. M. Schönenberger, H. Ewald, D. O. Lawson, D. Papola, R. Rohner, et al. Introducing the Library of Guidance for Health Scientists (LIGHTS): A Living Database for Methods Guidance. JAMA Netw Open. 2023;6(2):e2253198. Epub 20230201. DOI: 10.1001/jamanetworkopen.2022.53198.

APPENDIX C: Narrative synthesis of all topics for which a need for concretisation is present

1. Sterne JAC, et al. RoB 2: a revised tool for assessing risk of bias in randomised trials. BMJ. 2019 Aug 28;366:l4898. doi: 10.1136/bmj.l4898. PMID: 31462531. [↑](#footnote-ref-1)
2. Sterne JA, et. al. ROBINS-I: a tool for assessing risk of bias in non-randomised studies of interventions. BMJ. 2016 Oct 12;355:i4919. doi: 10.1136/bmj.i4919. PMID: 27733354; [↑](#footnote-ref-2)
3. Institute of Health Economics (IHE). Quality Appraisal of Case Series Studies Checklist. Edmonton (AB): Institute of Health Economics; 2014. Available from: http://www.ihe.ca/research-programs/rmd/cssqac/cssqac-about [↑](#footnote-ref-3)
4. Glasziou P, Chalmers I, Rawlins M, McCulloch P: When are randomised trials unnecessary? Picking signal from noise. BMJ 2007, 334(7589):349. [↑](#footnote-ref-4)
5. Requirements refer to a „focused search“ [↑](#footnote-ref-5)
6. Sun X, et al. Credibility of claims of subgroup effects in randomised controlled trials: systematic review. *BMJ* 2012; **344**: e1553.

   Sun X, et al. Is a subgroup effect believable? Updating criteria to evaluate the credibility of subgroup analyses. BMJ 2010; 340: c117. https://dx.doi.org/10.1136/bmj.c117. [↑](#footnote-ref-6)
7. Nikolakopoulou A, et al. CINeMA: An approach for assessing confidence in the results of a network meta-analysis. PLoS Med. 2020 Apr 3;17(4):e1003082. doi: 10.1371/journal.pmed.1003082. PMID: 32243458; PMCID: PMC7122720. [↑](#footnote-ref-7)
8. <https://www.riskofbias.info/welcome/rob-me-tool>, assessed on 01.05.2024 [↑](#footnote-ref-8)
